# Peroxisome Metabolism Contributes to PIEZO2-Mediated Mechanical Allodynia

**DOI:** 10.3390/cells11111842

**Published:** 2022-06-04

**Authors:** Yi Gong, Fiza Laheji, Anna Berenson, April Qian, Sang-O Park, Rene Kok, Martin Selig, Ryan Hahn, Reza Sadjadi, Stephan Kemp, Florian Eichler

**Affiliations:** 1 Department of Neurology, Massachusetts General Hospital, Harvard Medical School, Boston, MA 02114, USA; ygong4@mgh.harvard.edu (Y.G.); fiza.l@hotmail.com (F.L.); annaber@bu.edu (A.B.); aqian@mgh.harvard.edu (A.Q.); sangopark@college.harvard.edu (S.-O.P.); mselig@partners.org (M.S.); rhahn7805@gmail.com (R.H.); rseyedsadjadi@mgh.harvard.edu (R.S.); 2 Laboratory Genetic Metabolic Diseases, Department of Clinical Chemistry, Amsterdam University Medical Center, Amsterdam Gastroenterology Endocrinology Metabolism, University of Amsterdam, 1105 Amsterdam, The Netherlands; rene.kok93@gmail.com (R.K.); s.kemp@amsterdamumc.nl (S.K.); 3 Department of Pediatric Neurology, Emma Children’s Hospital, Amsterdam University Medical Center, Amsterdam Neuroscience, University of Amsterdam, 1105 Amsterdam, The Netherlands

**Keywords:** dorsal root ganglion (DRG), X-linked adrenoleukodystrophy (X-ALD), adrenomyeloneuropathy (AMN), mechanical hypersensitivity, pain, PIEZO2, peroxisomes, satellite glial cells (SGCs), glial fibrillary acidic protein (GFAP), RNA-seq, allodynia

## Abstract

Mutations in the peroxisomal half-transporter ABCD1 cause X-linked adrenoleukodystrophy, resulting in elevated very long-chain fatty acids (VLCFA), progressive neurodegeneration and an associated pain syndrome that is poorly understood. In the nervous system of mice, we found ABCD1 expression to be highest in dorsal root ganglia (DRG), with satellite glial cells (SGCs) displaying higher expression than neurons. We subsequently examined sensory behavior and DRG pathophysiology in mice deficient in ABCD1 compared to wild-type mice. Beginning at 8 months of age, *Abcd1^−/y^* mice developed persistent mechanical allodynia. DRG had a greater number of IB4-positive nociceptive neurons expressing PIEZO2, the mechanosensitive ion channel. Blocking PIEZO2 partially rescued the mechanical allodynia. Beyond affecting neurons, ABCD1 deficiency impacted SGCs, as demonstrated by high levels of VLCFA, increased glial fibrillary acidic protein (GFAP), as well as genes disrupting neuron-SGC connectivity. These findings suggest that lack of the peroxisomal half-transporter ABCD1 leads to PIEZO2-mediated mechanical allodynia as well as SGC dysfunction. Given the known supportive role of SGCs to neurons, this elucidates a novel mechanism underlying pain in X-linked adrenoleukodystrophy.

## 1. Introduction

X-linked adrenoleukodystrophy (X-ALD) is a debilitating progressive neurological disorder with pain often representing a persistent chronic symptom impacting the quality of life of patients [1,2,3]. X-ALD is caused by mutations in the *ABCD1* gene located on chromosome Xq28 [4]. The *ABCD1* gene encodes a peroxisomal ATP-binding cassette (ABC) half-transporter, which shuttles CoA-activated very long-chain fatty acids (VLCFA), such as C26:0-CoA and C24:0-CoA, into peroxisomes for degradation by beta-oxidation. This accumulation of saturated VLCFA is considered the biochemical hallmark of X-ALD and peroxisome dysfunction at large [5,6]. The most common phenotype of X-ALD is adrenomyeloneuropathy (AMN), a degenerative axonopathy that involves the ascending and descending tracts of the spinal cord [7,8,9]. In X-ALD, this form of myeloneuropathy affects almost all men and 80% of women [2,10]. Alongside gait disturbances and balance problems, neuropathic pain manifests as symmetrical, predominantly distal, burning or stabbing pain often requiring the use of analgesics. As a group of cell bodies responsible for transmitting sensory messages from various receptors to the CNS, the dorsal root ganglia (DRG) are thought to play a central role. In AMN these DRG neurons undergo atrophy, with ultrastructural demonstration of abnormal mitochondria but no appreciable neuronal loss [11,12].

Although pain and peripheral neuropathy are well reported manifestations in AMN, there is no detailed research on sensory change and the pathophysiology of early changes in the peripheral nervous system. Hence, in the present study, we characterize the sensory behavior in *Abcd1^-/y^* mice and address the underlying cellular and molecular mechanisms pertinent to the pathophysiology of DRG.

## 2. Materials and Methods

### 2.1. Animals

Congenic C57BL/6 *Abcd1^−/y^* and wild-type (WT) C57BL/6 mice were acquired from the Jackson Laboratory [13]. *Abcd1^−/y^* mice were backcrossed onto a pure C57/B6 background over six generations. They were then bred from homozygous founders, and occasionally genotyped according to the protocol provided by the Jackson Laboratory. All mice were kept in the animal housing facility of the Massachusetts General Hospital (MGH) Center for Comparative Medicine, had ad libitum access to water and standard rodent food, and were kept on a 12 h light–dark cycle.

### 2.2. Mouse Hot Plate Testing

Each mouse was habituated at 37 °C on the hot plate for 1 min and then tested with the temperature set to 50 °C and 52 °C. Mice were removed from the hot plate as soon as stereotypic behavior indicating discomfort was noted, or after 20 s had elapsed, so that no damage was inflicted upon the paws. The timing of stereotypic behavior was observed, recorded, and used for analysis.

### 2.3. Von Frey Filament Testing Mechanical Sensitivity

Rodents exhibit a paw withdrawal reflex when the paw is unexpectedly touched. Mechanical sensitivity was assessed with Von Frey filaments presented in order of increasing stiffness and usually started with nominal force 1 (North coast medical, CA, USA). These filaments were applied to the underside of the paw after the mouse was settled into a comfortable position within a restricted area that has a perforated floor. If the mouse responded by withdrawing, flinching, or licking the paw 2 times out of 10, the examiner moved to the next finer (lower number) filament [14]. Examination proceeded in this manner until the mouse did not respond. The last filament to which the mouse responded was defined as the mechanical threshold.

### 2.4. Brush Assay Test

Similar to the Von Frey test, a soft paint brush was gently applied to the plantar surface of the hind paw and the percentage of withdrawals out of 5 trials was counted.

### 2.5. Satellite Glial Cell Culture

Satellite glial cells (SGCs) were cultured following the protocol described by Poulsen [15]. Briefly, DRG tissues from 1–2month old mice were dissected and incubated in 5 mg/mL collagenase IV for 45 min at 37 °C. Digested tissues were further mechanically dispersed with a glass pipette. The collagenase solution was centrifuged, and the pellet was resuspended in 1 mL 0.125% trypsin and incubated for 10 min followed by another round of mechanical dissociation before the suspension was centrifuged once more. The cell pellet was resuspended in DMEM/F12 containing 10% FBS and 1% pen/strep and cultured at a humidified 5% CO_2_ incubator (37 °C). Cell medium was replaced after 2 h and 24 h and then every 2 days until confluency.

### 2.6. Immortalized Rodent DRG Neuron Culture and Lipid Supplementation

Immortalized rat DRG neurons were obtained from the GRCF Biorepository & Cell Center at the John Hopkins School of Medicine (thanks to Dr. Ahmet Hoke) [16]. Cells were maintained in neurobasal medium containing 10% FBS, 0.2% glucose, Glutamax, and B27. Differentiation was performed by supplementing medium with 75 μM forskolin, 25 ng/mL NGF, and 25 ng/mL GDNF. DRG neurons were differentiated for 3 days and then treated with lipids (5 μM of C16:0, C24:0 or C26:0 free fatty acids (Sigma-Aldrich, St. Louis, MO, USA) dissolved in ethanol) for 3 days.

### 2.7. Western Blotting

Tissue and cell lysates were prepared by using RIPA buffer (Sigma-Aldrich, St. Louis, MO, USA) with 1% Halt Protease and Phosphatase Inhibitor Cocktail (Roche, Indianapolis, IN, USA). Protein samples were separated on NuPAGE 4–12% Bis-tris gels (Invitrogen, Carlsbad, CA, USA) and transferred on PVDF membranes. Membranes were blocked with 5% non-fat milk in PBS containing 0.05% Tween 20 and probed with antibodies against ABCD1 (Abcam, ab197013, 1:5000), GFAP (Abcam, ab7260, 1:1000), CGRP (Santa Cruz, sc57053, 1:500) and peripherin (Merke, MAB1527, 1:500). Anti-β-ACTIN (Santa Cruz, SC-47,778, 1:1000) and anti-GADPH (Santa Cruz, sc3223, 1:1000) were used as a protein loading control. Membrane protein signals were prepared by using SuperSignal West Pico Chemiluminescent Substrate (Thermo, Rockford, IL, USA) after incubation with HRP-conjugated secondary antibodies.

### 2.8. Immunofluorescence Staining and Confocal Microscopy Imaging

Sections of DRG tissue (14 μm) were cut at −23 °C using cryostat (Leica). Sections were stained with anti-ABCD1 (Abcam, ab197013, 1:200), anti-GFAP (Dako, Z0334, 1:500), anti-Neurofilament (SMI32) (Biolegend, 801,701, 1:1000), peripherin (Abcam, ab1530, 1:500, Cambridge, UK), CGRP (Abcam, ab42072, 1:1000), C-caspase3 (Cell signaling, 9661, 1:400), and anti-NeuN (Abcam, ab104224, 1:1000) antibodies separately or in combination. We used an anti-ABCD3 antibody to visualize peroxisomes (Abcam, ab3421, 1:1000). Isolectin GS-IB4 Alexa Fluor™ 488 Conjugate from Griffonia simplicifolia (Invitrogen) was prepared at 1mg/mL stock solution and applied to slides at 1:500 dilution when needed. The slides were imaged by confocal laser microscope. Fluorescence intensity was quantified by Image J (Wayne Rasband, NIH).

### 2.9. Lipid Analysis

DRG tissues or cultured SGCs were harvested and then analyzed by electrospray ionization mass spectrometry (ESI-MS), as described previously [6]. Absolute values of C24:0 and C26:0 in ng/mg protein were reported.

### 2.10. Transcriptome Sequencing

RNA from freshly dissected mouse DRG tissue and cultured SGCs was extracted using RNeasy mini kit (Qiagen). Transcriptome sequencing was performed by Macrogen. Analyses were performed on 6 WT and *Abcd1^−/y^* paired-end samples. Overall sequenced reads quality, total bases, total reads, GC (%), and basic statistics were calculated. The splice-aware alignment program STAR (http://bioinformatics.oxfordjournals.org/content/early/2012/10/25/bioinformatics.bts635 (accessed on 1 January 2017 and 1 August 2017)) was used to map sample sequencing reads (from .fastq files) to a Mus musculus (mm10 build) reference genome file. Gene expression counts were calculated using the program HTSeq (http://www-huber.embl.de/users/anders/HTSeq/doc/overview.html (accessed on 1 January 2017 and 1 August 2017) based on a current Ensembl annotation for mm10. The R package “edgeR” was employed to make differential gene expression calls from these counts per the following criteria: Gene expression was considered upregulated if log2 FC > +1 or downregulated if the log2 FC < −1 (FC = fold-change of average RPKM) with respect to the conditions being compared. Differentially expressed genes were identified through volcano plot filtering.

### 2.11. Enrichment Analysis

The differentially expressed genes (DEGs) selected by the bioinformatical criteria above were used for functional analysis by the Metacore software. Process network, toxicity network, and gene ontology (GO) process enrichment analyses were performed.

### 2.12. Quantitative Real-Time Reverse-Transcription PCR

Total RNA was isolated by using Qiagen RNeasy Mini Kit (Qiagen). First-strand cDNA synthesis used 100 ng random primer (Invitrogen, Carlsbad, CA, USA), 1.0 μg total RNA, 10 mM dNTP and 200 units of reverse transcriptase (Invitrogen, Carlsbad, CA, USA) per 20 μL reactions. PCRs were performed in duplicates in a 25 μL final volume by using SYBR Green master mix from applied biosystems (Warrington, UK), and the data were analyzed by calculating the delta Ct value between testing gene and internal control. Primers used in the experiment were as described in Supplementary Table S1.

### 2.13. Intrathecal Injection of D-GsMTx4

Briefly, 8–10-month-old male C57BL/6 *Abcd1^−/y^* mice were put under anesthesia by isoflurane. After the skin over the lumbar region was shaved and cleaned, a 3~4 cm mid-sagittal incision was made through the skin exposing the muscle and spine. A catheter was inserted between L4–L5 spine region and attached to a gas-tight Hamilton syringe with a 33-gauge steel needle. Then, 25 μM D-GsMTx4 dissolved in 10 μL saline were slowly injected at a rate of 5 μL/min with saline injection as control.

### 2.14. Statistical Analysis

Results were expressed as means ± SEM and analyzed for statistical significance by ANOVA followed by Bonferroni testing for multiple comparisons among experimental groups (IBM SPSS Statistics for Windows, Version 26.0. Armonk, NY, USA: IBM Corp.). Two-tailed *t*-tests were used for the comparison of WT and *Abcd1^−/y^* groups. A *p* < 0.05 was considered statistically significant.

## 3. Results

### 3.1. ABCD1 Expression in DRG Tissue

**Figure 1:**
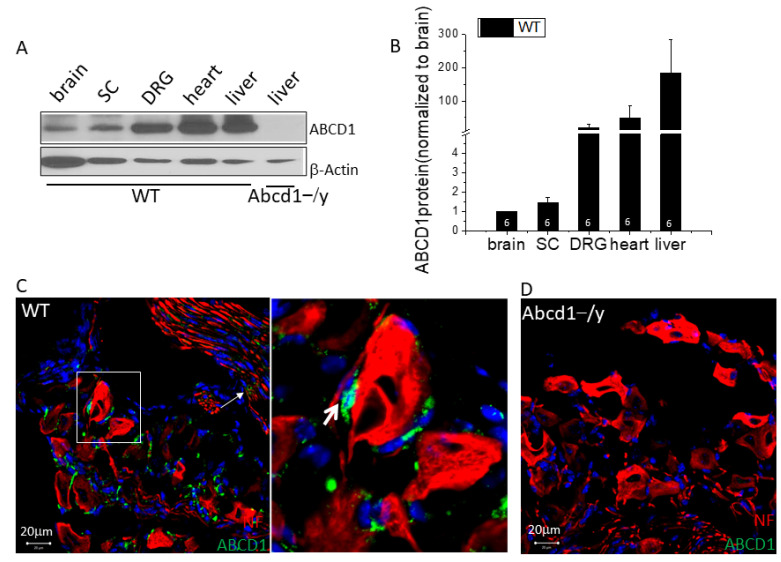
Within the nervous system, ABCD1 protein is highest in satellite glial cells (SGC) of dorsal root ganglia (DRG). (**A**) Representative Western blot image showing ABCD1 protein expression in different mouse organs with *Abcd1^−/y^* mouse DRG tissue as negative control. SC = spinal cord (**B**) ABCD1 protein level in different organs of WT mice was quantified by image J with β-ACTIN as loading control (*n* = 6). (**C**) Representative confocal images showing ABCD1 mainly expressed in SGCs (thick arrow) that surround DRG neurons; Schwann cells (thin arrow) surrounding axons also show ABCD1 expression. Magnification shows the close proximity of ABCD1 to the neuronal membrane surface. (**D**) In *Abcd1^−/y^* DRG, there is no ABCD1 detectable in SGCs. Bar = 20 μm. Results were expressed as means ± SEM.

Within the nervous system, DRG have higher ABCD1 expression than the brain and spinal cord (Figure 1A,B). The ABCD family member with closest homology and function to ABCD1 is ABCD2 [17,18]. Interestingly, ABCD2 expression in DRG is strikingly lower compared to that in the brain and spinal cord, possibly contributing to the vulnerability of DRG in the absence of ABCD1 (Figure S1). Within DRG, ABCD1 protein localized largely to SGCs that surround the neurons, whereas the neurons themselves had barely detectable ABCD1 protein (Figure 1C, thick arrow). Some ABCD1 protein was also detected around the axons (Figure 1C, thin arrow), pointing to Schwann cell expression of ABCD1. In addition, ABCD1 expression at the embryonic stage (E13.5), when glial cells are less developed, was much lower but increased significantly postnatally (Figure S2).

### 3.2. Nociceptive Behavior in Abcd1^−/y^ Mice

**Figure 2:**
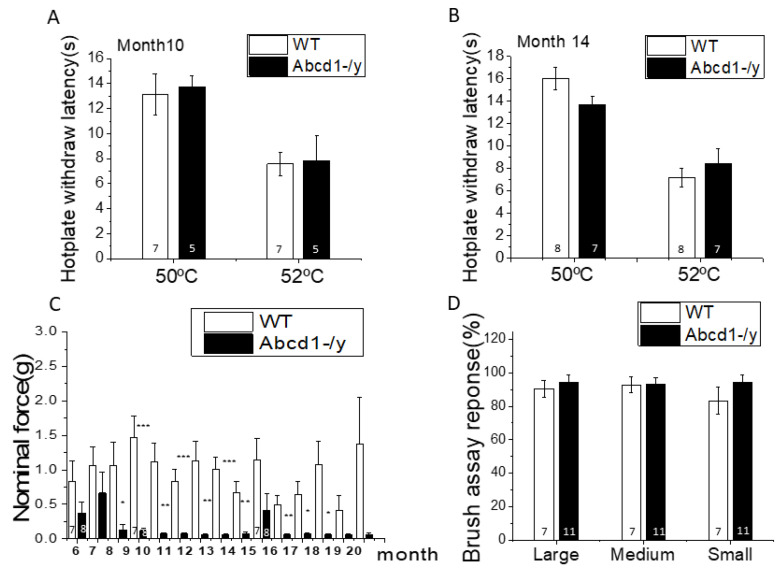
*Abcd1^−/y^* mice exhibit mechanical but not thermal hypersensitivity. (**A**) Thermal sensitivity of *Abcd1^−/y^* mice measured by hotplate testing at 10 months (*n* = 7 for WT, *n* = 5 for *Abcd1^−/y^*). (**B**) Thermal sensitivity of *Abcd1^−/y^* mice measured by hotplate testing at 14 months (*n* = 8 for WT, *n* = 7 for *Abcd1^−/y^*). (**C**) Mechanical sensitivity of Abcd1-/y mice measured by Von Frey testing from 6 months until 20 months of age (*n* = 7 for WT, *n* = 8 for *Abcd1^−/y^*). (**D**) Brush assay in 9–10-month-old mice (*n* = 7 for WT, *n* = 11 for *Abcd1^−/y^*). Results were expressed **as** means ± SEM. * *p* < 0.05, ** *p* < 0.01, and *** *p* < 0.001 as compared with WT control.

Peripheral neuropathy in *Abcd1^−/y^* mice is known to manifest as motor impairment around 15 months of age. To characterize the sensory behavior of *Abcd1^−/y^* mice, we monitored sensation beginning at 6 months of age by using hot plate (thermosensitivity) and Von Frey (mechanical sensitivity) testing. On hot plate testing, no significant difference between WT and *Abcd1^−/y^* was found at either 10 or 14 months of age (Figure 2A,B). On the other hand, mechanical hypersensitivity developed around 8 months of age, and this hypersensitivity persisted until 20 months of age (Figure 2C). *Abcd1^−/y^* mice showed consistent acute sensitivity to gentle touch with small filaments which WT mice normally do not respond to. Although aging contributed to some variability, the early mechanical hypersensitivity was reproducible in separate sets of studies performed by different technicians over the years (Figure S3), indicating persistent peripheral sensory dysfunction in several generations of *Abcd1^−/y^* mice. Further brush assays on 9–10-month-old mice validated the hypersensitivity of *Abcd1^−/y^* mice, although not to the same degree as the Von Frey assay (Figure 2D).

### 3.3. Peroxisomal Dysfunction in Abcd1^−/y^ Mice

**Figure 3:**
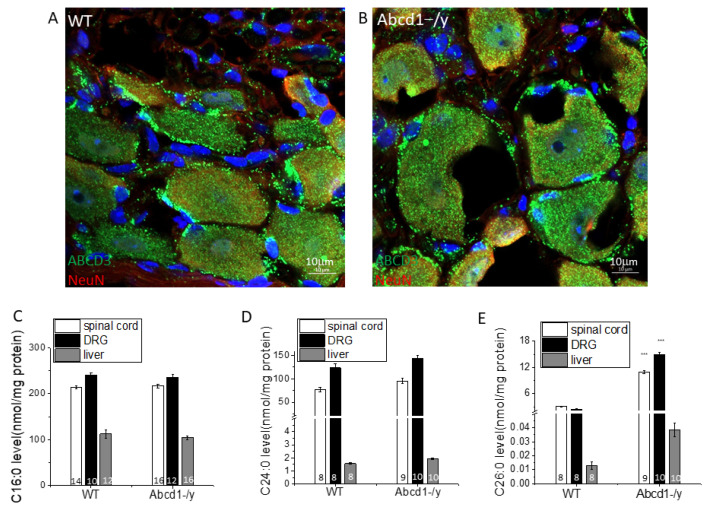
Peroxisome dysfunction in *Abcd1^−/y^* mice leads to highest accumulation of very long chain fatty acids (VLCFA) within DRG. Peroxisomes are present in both SGCs and neurons as evident by ABCD3 staining. They are present in greater abundance within SGCs in both WT (**A**) and *Abcd1^−/y^* (**B**), Bar = 10 μm. The VLCFA C24:0 (**D**) and C26:0 (**E**) but not C16:0 (**C**) were higher in *Abcd1^−/y^* mouse organs compared to wild-type measured by electrospray ionization mass spectrometry (ESI-MS) and reported as nmol/mg protein (*n* = 8–14 for WT and *n* = 9–16 for *Abcd1^−/y^*, numbers are labeled in each Figure). Results were expressed as means ± SEM. *** *p* < 0.001 as compared with WT control.

While SGCs appear to be enriched in peroxisomes as visualized by ABCD3(PMP70) staining, an extensively used peroxisome marker, the distribution of peroxisomes in both SGCs and neurons showed little difference between *Abcd1^−/y^* and WT mice (Figure 3A,B). Lipid analysis revealed that *Abcd1^−/y^* DRG has higher VLCFA (C26:0 and C24:0) accumulation than spinal cord and liver but no increase in short chains such as C16:0 (Figure 3C). Compared to the 3.7-fold increase of the C26:0 level in *Abcd1^−/y^* spinal cord and the 2.9-fold increase in liver, DRG reached a 5.9-fold increase in C26:0 over WT. In addition, the absolute C26:0 level in the *Abcd1^−/y^* nervous system (10.9 nmol/mg protein in the spinal cord and 14.8 nmol/mg protein in DRG) was around 300 times higher than that in the liver (0.04 nmol/mg protein), implying the importance of ABCD1 protein to nerve function overall (Figure 3D,E).

### 3.4. Neuron Subtype Distribution in DRG

Sensory neurons can be broadly subdivided into functional classes based on their stimulus response. To determine whether there was any difference in neuron subtype distribution in *Abcd1^−/y^* mice, we performed neurofilament H(NF-H) and peripherin staining in mouse DRG tissue and quantified the percentage of NF-positive and peripherin-positive neurons. Neurofilament-H primarily labels large-sized neurons mediating mechanoreception and proprioception, while peripherin primarily labels small neurons responsible for pain and temperature sensation and can be further divided into calcitonin gene-related peptide (CGRP)-positive and isolectin IB4-binding neurons (IB4+) [19].

**Figure 4:**
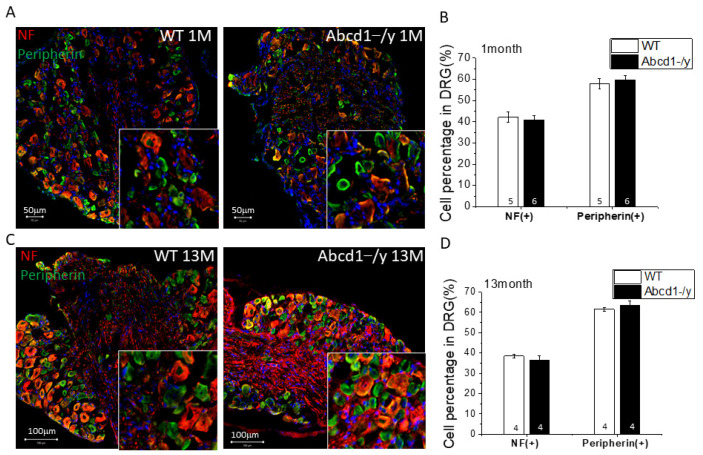

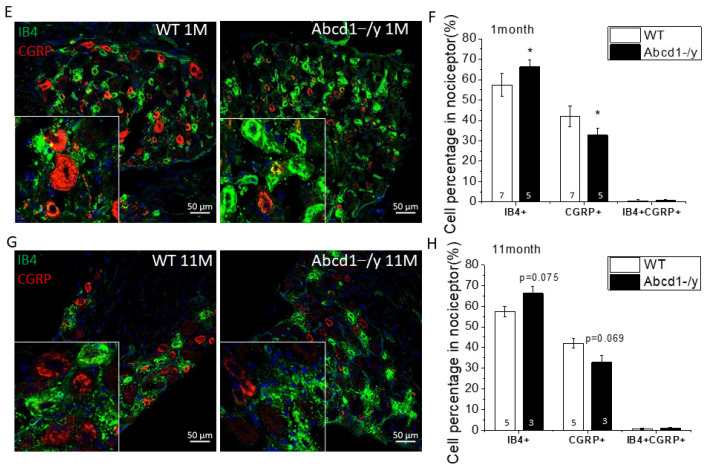

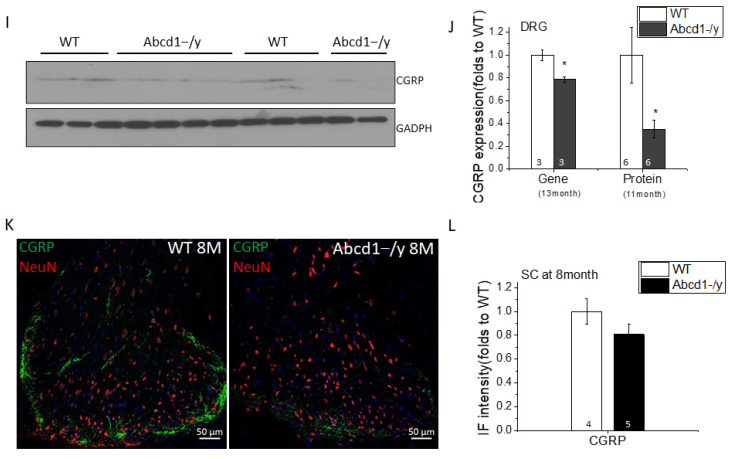
Dorsal root ganglia of *Abcd1^−/y^* mice reveal an increase in IB4-positive mechanical nociceptors. (**A**) Representative confocal images showing NF (red) and peripherin (green) staining in 1-month-old mouse DRG. Bar= 50 μm. (**B**) NF-positive and peripherin-positive neurons were quantified by image J in 1-month-old mouse DRG (*n* = 5 for WT and *n* = 6 for *Abcd1^−/y^*). (**C**) Representative confocal images showing NF (red) and peripherin (green) staining in 13-month-old DRG. Bar = 100 μm. (**D**) NF-positive and peripherin-positive neurons were quantified by image J in 13-month-old mouse DRG (*n* = 4 for WT and *n* = 4 for *Abcd1^−/y^*. (**E**) Representative confocal images showing CGRP (red) and IB4-conjugate (green) staining in 1-month-old mouse DRG. (**F**) CGRP-positive and IB4-positive neurons were quantified by image J in 1-month-old mouse DRG (*n* = 7 for WT and *n* = 5 for *Abcd1^−/y^*). (**G**) Representative confocal images showing CGRP (Red) and IB4-conjugate (green) staining in 11-month-old mouse DRG. (**H**) CGRP-positive and IB4-positive neurons were quantified by image J in 11-month-old mouse DRG (*n* = 5 for WT and *n* = 3 for *Abcd1^−/y^*). (**I**) Western blot images showing CGRP expression in *Abcd1^−/y^* DRG (*n* = 6 for each). (**J**) Quantification of CGRP gene and protein expression in *Abcd1^−/y^* DRG. (**K**) Representative confocal images showing NeuN (red) and CGRP (green) staining in 8-month-old mouse spinal cord dorsal horn. (**L**) Quantification of CGRP immunostaining intensity in spinal cord dorsal horn (*n* = 4 for WT and *n* = 5 for *Abcd1^−/y^*). Results were expressed as means ± SEM. * *p* < 0.05 as compared with WT control.

No significant difference was found in the distribution of NF-positive and peripherin-positive neurons between WT and *Abcd1^−/y^* mice at either 1 month (Figure 4A,B) or 13 months of age (Figure 4C,D). In addition, cleaved capase3 (C-caspase3) staining in DRGs of 15-month-old mice did not reveal apoptosis (Figure S4), suggesting no clear neuronal loss, as previously described in humans [12]. However, further neuronal subtyping revealed a decrease in the percentage of CGRP-positive but an increase in IB4-positive neurons at both 1 month (Figure 4E,F) and 11 months (Figure 4G,H) of age. The percentage of neurons that were both IB4- and CGRP-positive was only 1–2%, suggesting a clear segregation of these two types of nociceptors (Figure 4E–H). Further analysis of CGRP in DRG revealed significantly reduced CGRP expression at both the mRNA and protein levels, in line with the changes in cell proportion (Figure 4I,J). Since the CGRP-positive DRG neurons mainly project into the spinal cord dorsal horn, we assessed the CGRP expression at DRG axon terminals in the spinal cord. Consistent with the reduction of CGRP in DRG soma, CGRP at the spinal cord dorsal horn was also decreased by around 20% (Figure 4K,L).

### 3.5. DRG Transcriptomic Analysis

Following transcriptomic analysis, 15,474 genes were successfully mapped and identified from RNA-seq. In total, 161 differentially expressed genes (DEGs) were filtered out between WT and *Abcd1^−/y^* DRG at |log2 FC| > 1 threshold, which consisted of 80 upregulated genes and 81 downregulated genes (Figures S5 and S6). Enrichment analysis by process network identified several pathways potentially linked to the sensory changes, including those involved in neuropeptide signaling and potassium transport (Table 1). The toxicity network analysis revealed pathways relevant to neuropeptide signaling and transmission of nerve impulses as well as opioid, galanin, and neurotensin receptors (Table S2). Enrichment analysis by GO processes also identified genes involved in responses to external stimuli and regulation of responses to biotic stimuli (Table S3). All these results pointed to a dysregulated ion transport and disturbance in neural signaling transmission, which could potentially contribute to mechanical hypersensitivity.

**Figure 5:**
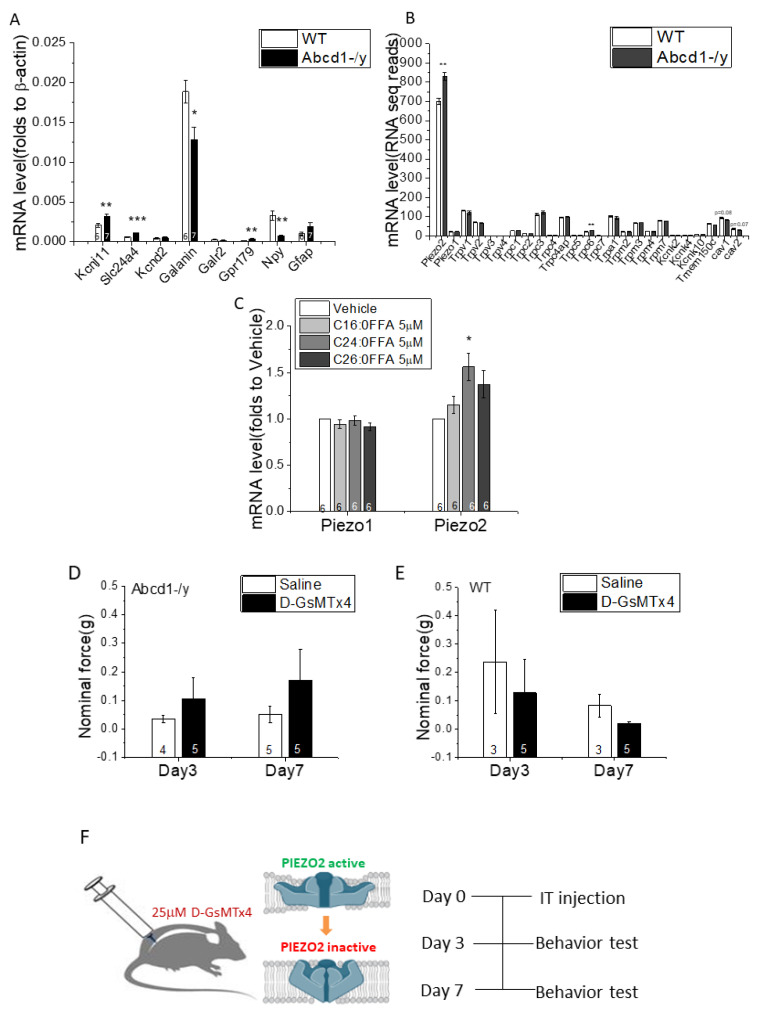
PIEZO2 is highly expressed in dorsal root ganglia of *Abcd1^−/y^* mice and blocking the mechanosensitive channel partially rescues mechanical allodynia. (**A**) qPCR verification of selected genes enriched in RNA−seq that are involved in key neuronal functions (*n* = 6 for WT and *n* = 7 for *Abcd1^−/y^*). (**B**) Profiling of mechanosensitive channel genes and caveolin genes from RNA-seq data. (**C**) Impact of lipids upon *Piezo1* and *Piezo2* gene expression in differentiated rat DRG neurons. Changes in mechanical sensitivity in (**D**) *Abcd1^−/y^* and (**E**) WT mice after intrathecal (IT) delivery of PIEZO2 blocker D-GsMTx4 (25 μM; sensory thresholds assessed by Von Frey testing). (**F**) Schematic illustration of D-GsMTx4 blocking of PIEZO2 channels by IT delivery. Results were expressed as means ± SEM. * *p* < 0.05, ** *p* < 0.01, and *** *p* < 0.001 as compared with WT control.

To confirm the accuracy of the RNA-seq results, we next used qPCR to assess several genes crucial to regulating neuronal ion transport and signaling transmission. *Kcnj11*, *Slc24a4*, and *Gpr179* were significantly upregulated and *Galanin* and *Npy* significantly downregulated. Moderate increases of *Gfap* suggested by RNA-seq data were also verified (Figure 5A). We also profiled a cluster of mechanosensitive channel genes: *Piezo2* and *Trpc6* were significantly upregulated albeit not reaching the |log2 FC| > 1 threshold, while two genes responsible for caveolae formation on cellular membranes (*Cav1* and *Cav2*) were downregulated (Figure 5B). Interestingly, lipid treatment with saturated VLCFA led to significantly increased Piezo2 but not Piezo1 mRNA expression, which is in line with RNA-seq data and suggested that the aberrant lipids could be contributing to the Piezo2 upregulation.

With the high abundance of PIEZO2 expression in DRG and its known role in mediating mechanosensitivity, we tested whether PIEZO2 contributed to the mechanical hypersensitivity of *Abcd1^−/y^* mice by performing intrathecal delivery of the PIEZO2-blocking chemical D-GsMTx4 (Figure 5F). Initially after intrathecal delivery *Abcd1−/y* mice remained hypersensitive, but by 7 days, the D-GsMTx4-injected mice showed a reduction in hypersensitivity compared to the saline-injected group (Figure 5D–E). Interestingly, *Abcd1−/y* mice after D-GsMTx4 injection also showed reduced balance time on the rotarod test 7 days post injection (data not shown). Prior reports showed that conditional knockout of PIEZO2 in mouse DRG led to defective proprioception, also manifesting as reduced latency on the rotarod test [20].

### 3.6. Increased GFAP Expression in DRG Tissue in the Absence of ABCD1

**Figure 6:**
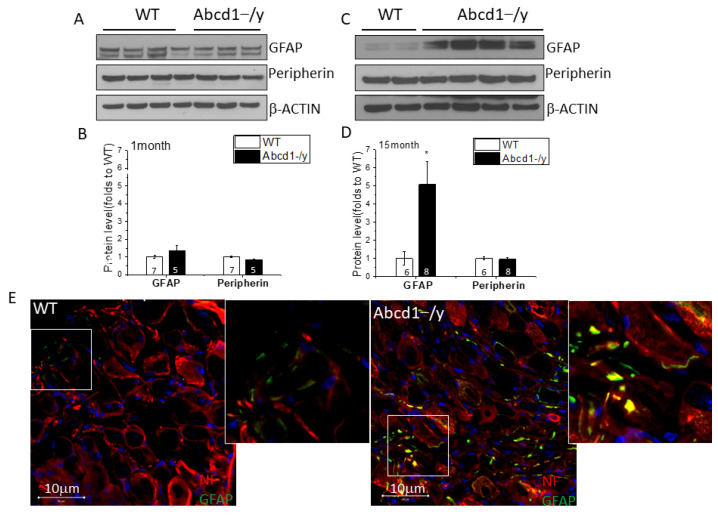
Increased GFAP expression occurs early in *Abcd1^−/y^* mouse DRG. (**A**) Representative western blot images showing GFAP expression in 1-month-old mouse DRG tissue with peripherin as comparison. (**B**) Quantification of GFAP protein level in 1-month-old mouse DRG tissue with β−ACTIN as loading control (*n* = 7 for WT and *n* = 5 for *Abcd1^−/y^*). (**C**) Representative Western blot images showing GFAP expression in 15-month-old mouse DRG tissue with peripherin as comparison. (**D**) Quantification of GFAP protein level in 15-month-old mouse DRG tissue with β−ACTIN as loading control (*n* = 6 for WT and *n* = 8 for *Abcd1^−/y^*). (**E**) Representative confocal images showing increased GFAP staining (green) in 15-month-old *Abcd1^−/y^* mouse DRG. Results were expressed as means ±SEM. * *p* < 0.05 as compared with WT control.

Aside from dysregulation in ion transport and neurotransmission, the DRG transcriptome data also suggested increased *Gfap* gene expression in *Abcd1^−/y^* DRG tissue. For verification, we compared GFAP protein expression between WT and *Abcd1^−/y^* DRG tissues at different ages. A significant increase (around 5-fold) of GFAP expression was detected in 15-month-old *Abcd1^−/y^* mouse DRG tissue. Surprisingly, a trend in increased GFAP was seen as early as 1 month of age (Figure 6A–D). Further immunohistochemistry suggested that the increased GFAP signal was localized to glial cells surrounding neurons (satellite glial cells, SGCs) as well as some Schwann cells (Figure 6E, magnification). To understand whether glial cell proliferation was contributing here, we quantified the average SGC number and found no significant difference (Figure S7).

### 3.7. Increased GFAP Expression and VLCFA Accumulation in Abcd1^−/y^ SGCs

**Figure 7:**
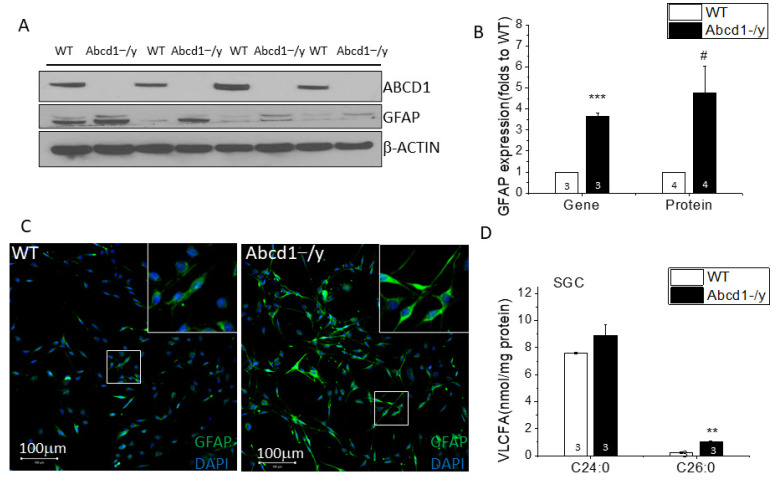
Gliosis and peroxisome dysfunction in satellite glial cells of *Abcd1^−/y^* mice. (**A**) Western blot images showing increased GFAP expression in primary cultured *Abcd1^−/y^* SGCs with β-ACTIN as loading control. ABCD1 expression confirmed knockdown in *Abcd1^−/y^* mice. (**B**) Quantification of GFAP gene (qPCR) and protein expression in *Abcd1^−/y^* SGCs (*n* = 4 for protein groups and *n* = 3 for gene groups). (**C**) Representative confocal images showing increased GFAP staining (green) in primary cultured *Abcd1^−/y^* SGCs. (**D**) Increased C26:0 and C24:0(nmol/mg protein) in primary cultured SGCs from *Abcd1^−/y^* mice (*n* = 3 for each group). Results were expressed as means ± SEM. # *p* < 0.05, ** *p* < 0.01, and *** *p* < 0.001 as compared with WT control.

To study the functional consequences of ABCD1 deficiency in SGCs, we isolated SGCs from mouse DRG tissue and performed in vitro culture as well as subsequent protein and biochemical analysis. Interestingly, the SGCs isolated from 1–2 month-old *Abcd1^−/y^* DRG tissue showed significantly increased GFAP at the gene and protein level (Figure 7A–C), implying early SGC activation due to ABCD1 deficiency. Further lipid analysis revealed that *Abcd1^−/y^* SGCs had an approximate 4.5-fold increase of C26:0 fatty acid and 1.2-fold increase of C24:0 fatty acid (Figure 7D), in line with the overall C26:0 increase in DRG tissue.

### 3.8. SGC Transcriptomic Analysis

**Figure 8:**
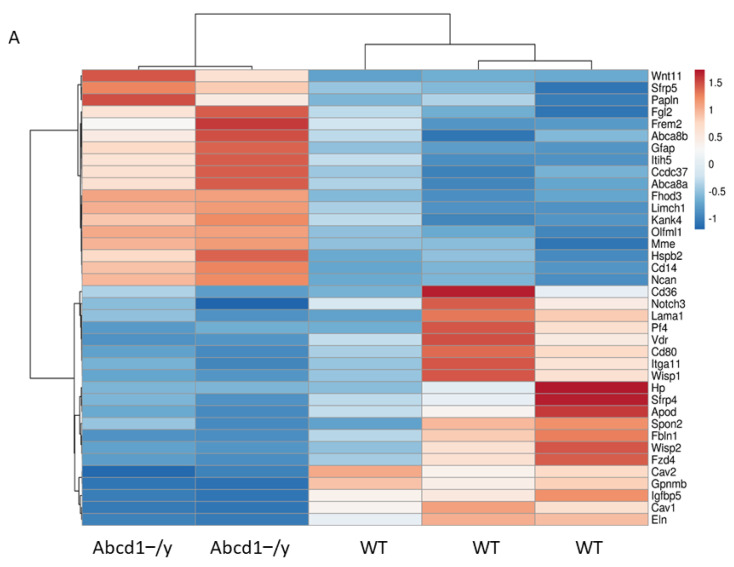

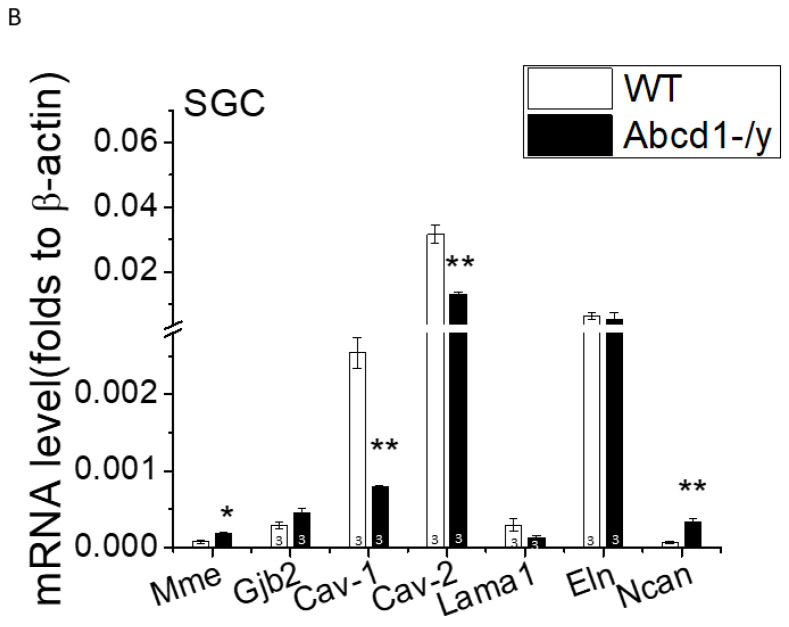
Activated satellite glial cells alter genes potentially affecting mechanical properties of neuronal membranes. (**A**) Heat map showing the hierarchical clustering of genes related to cell adhesion and cell matrix interaction as well as lipid and vesicle membrane transportation in *Abcd1^−/y^* SGCs at |log2 FC| > 1 threshold. (**B**) qPCR verification of selected genes enriched in RNA-seq that are involved in membrane mechanical properties (*n* = 3 for each group). Results were expressed as means ± SEM. * *p* < 0.05 and ** *p* < 0.01 as compared with WT control.

Transcriptomic analysis in isolated SGC mapped and identified 12,243 genes successfully from RNA-seq. In total, 196 differentially expressed genes (DEGs) (85 upregulated genes and 111 downregulated genes) were identified between WT and *Abcd1^−/y^* SGCs at |log2 FC| > 1 threshold (Figure S8). In the enrichment analysis by process network, pathways involving cell adhesion and cell matrix interaction emerged and involved the following genes: membrane metalloendopeptidase (*Mme*) (up), neurocan (*Ncan*) (up), elastin (*Eln*)(down), laminin (*Lama1*) (down), caveolin1,2 (*Cav-1,2*) (down) (Table 2). Enrichment analysis by toxicity network also pointed to many cell adhesion–related pathways as well as lipid and vesicle transport which comprise CD36 (down), caveolin (down), SR-BI (down), HDL protein (down) and VDR (down) (Table S4). Enrichment analysis by GO processes discovered pathways related to responses to lipid perturbations, responses to external or biotic stimuli and regulation of cell motility (Table S5), which were in line with the pathways enriched in DRG transcriptomic analysis and of special importance to a metabolic disorder with mechanical hypersensitivity. We also selected several relevant genes for qPCR verification. Significant upregulation of the *Mme* and *Ncan* genes and downregulation of the *Cav-1* and *Cav-2* genes as well as trend changes in the *Lama1* and *Eln* genes matched the RNA-seq results well, suggesting reliability of the RNA-seq data (Figure 8).

## 4. Discussion

Mutations in *ABCD1* cause a chronic degenerative disease called adrenoleukodystrophy with associated pain impacting gait, balance, and sleep in affected patients and requiring the use of chronic analgesics [1,2,3,21]. Examining the expression pattern of *ABCD1* we found dorsal root ganglia (DRG) to have the highest levels of *ABCD1* gene and protein expression in the entire nervous system (Figure 1). We further found that mice deficient in ABCD1 (*Abcd1^−/y^*) developed mechanical allodynia by 8 months of age, preceding motor abnormalities (Figure 2). Transcriptomics and lipid analysis of the DRG revealed an upregulation of PIEZO2 and lipid perturbations in neighboring glia (Figure 5, Figure 6 and Figure 7, Graphic abstract).

As in wild-type mice, high levels of detectable ABCD1 protein are seen in normal human DRG neurons [11]. Autopsy specimens of adults with AMN deficient in ABCD1 show no evidence of cell death in DRG [11,12], but DRG neurons undergo atrophy with ultrastructural demonstration of abnormal mitochondria. Similarly, in *Abcd1^−/y^* mice we found oxidative stress and lipid perturbation but no neuronal cell loss.

Sensory neurons are subdivided into functional classes based on their stimulus response. While *Abcd1^−/y^* mice showed no difference in the distribution of mechanoreceptive and proprioceptive (NF-positive) versus nociceptive neurons (peripherin-positive), further subtyping revealed a decrease in CGRP-positive and an increase in IB4-positive nociceptors (Figure 4). Since the non-peptidergic IB4-positive CGRP-negative nociceptive neurons mainly mediate noxious mechanical stimulus [19], these changes could partially explain the mechanical hypersensitivity observed. Prior studies have reported that following trauma, nociceptive neurons in DRG can proliferate ipsilateral to the injury [22]. Satellite glial cells in DRG may also directly communicate with sensory neurons, releasing neural modulating factors or stimulating the sprouting of axons [23,24].

We next considered whether the preponderance of IB4-positive neurons may be due to aberrant metabolism during early development. Peroxisome dysfunction in DRG was evident in *Abcd1^−/y^* mice at a young age (Figure 3). Saturated VLCFA, such as C26:0, were elevated 5.9-fold in DRG compared to 3.7-fold and 2.9-fold in the liver and the spinal cord, respectively. Saturated VLCFA (C26:0) are known to lead to lipid-induced ER stress, as evidenced by an increase in XBP1 [25]. While VLCFA are known to be toxic to cells, it is not known how this lipid perturbation could lead to sensory dysfunction. To investigate the pathways affected, we pursued transcriptomic analysis of DRG.

Electrical excitation of peripheral somatosensory nerves is commonly a first step in the generation of pain and is usually controlled by an intricate set of ion channels that together produce a degree of excitation proportional to the strength of external stimulation [26]. In *Abcd1^−/y^* mice, solute carrier (SLC) transporters, responsible for trafficking various substrates, such as inorganic ions, amino acids, fatty acids, neurotransmitters, and saccharides [27], were upregulated (Figure 5). Most important to our study of mechanical allodynia, transcriptomic analysis revealed an increase in PIEZO2 expression as well as a reduction in membrane caveolae structure proteins (CAV1 and CAV2) in *Abcd1^−/y^* mouse DRG.

PIEZO2 acts as the major transducer of mechanical forces for touch sensation and provides diverse sensory functions within DRG neurons [20,28,29], including mediating tactile pain [30]. Significantly, IB4-positive neurons are rich in PIEZO2 compared to CGRP-positive neurons [20,31]. The increase of PIEZO2 gene expression matched the observed increase in IB4-positive neurons, possibly accounting for the increased mechanical sensitivity in *Abcd1^−/y^* mice. However, an increase of mechanosensitive channels (MSC) such as PIEZO2 could increase currents independent of cell number. Aberrant lipids could further impact PIEZO2-mediated mechanical allodynia, as VCLFA treatment increased PIEZO2 in cultured rat DRG neurons and tension within the membrane bilayer is also known to activate MSC [32]. Caveolae, the membrane invaginations of lipid raft domains, regulate mechanosensitive channel function by modulating membrane tension, and reducing caveolins can increase MSC currents, such as those in PIEZO channels, supporting the key roles of these proteins in the development of mechanical hypersensitivity [33,34]. The highly positively charged peptide GsMTx4 inhibits MSC gating by associating with the cell membrane and buffering tension transmission to the channels [35]. By blocking PIEZO2 in *Abcd1^−/y^* mice using GsMTx4, we were able to partially rescue the mechanical allodynia. This corroborated the significance of PIEZO2 to the sensory abnormalities seen in *Abcd1^−/y^* mice but also suggested recalcitrant pathology within the *Abcd1^−/y^* DRG.

While humans have high levels of ABCD1 protein expressed in neurons themselves [36], mice have very high ABCD1 expression in the SGCs that surround the neuronal cell body and barely detectable levels in neurons. Of note, glial cells such as SGCs are rich in peroxisomes and the enzymes responsible for lipid synthesis and can thereby contribute to VLCFA accumulation and subsequent axonal degeneration [24,37]. SGCs had an approximate 4.5-fold increase of C26:0 fatty acid (Figure 7D), in line with the overall C26:0 increase in *Abcd1^−/y^* DRG tissue (5.9-fold). Although DRG neurons are the cells transmitting nociceptive signals, SGCs are now recognized as important to both axonal regeneration and the pathogenesis of chronic pain [24,38,39]. Interestingly, nerve injury elicits changes in the expression of genes related to fatty acid synthesis, and peroxisome proliferator-activated receptor (PPARα) signaling in SGCs appears essential to axonal regeneration [40]. Conversely, structural and biochemical changes in SGCs can cause aberrant connectivity between neighboring SGCs and sensory neurons and contribute to chronic pain. This can involve gliosis (i.e., proliferation), increased expression of GFAP (a marker for SGC activation), modulation of glutamate transporters and ion channels, and increases in purinergic and cytokine signaling [24,38,39,41].

In our study, an increase in GFAP in SGCs from 1-month-old *Abcd1^−/y^* mice suggests early glial activation, even before alterations in sensory behavior (Figure 6). Furthermore, transcriptomic analysis revealed aberrant cell adhesion, cell matrix interactions, and membrane transport which involved the downregulation of laminin (*Lama1*), elastin, and caveolins (*Cav1 and Cav2*), as well as upregulation of membrane metalloendopeptidase (*Mme*) and the chondroitin sulfate proteoglycan neurocan (*Ncan*), a member of the extracellular matrix of the nervous system (Figure 8). Lack of ABCD1 protein activates SGCs, thereby perturbing connectivity between neighboring neurons.

Prior studies examining the effects of saturated VLCFA upon model membranes predicted a disruptive effect upon cell membrane structure and function [10]. Membrane lipids have been found to impact mechanosensitive channels, as with stretching of the bilayer, the membrane and the local trans-bilayer pressure profile changes [36,42]. Pertinent to our finding of PIEZO2 dysfunction, the conformation of these ion channels is influenced by the stiffness of the lipid bilayer, thus producing gating movements to open the pore. While MSC are thus activated by bilayer tension in neuronal membranes, extracellular matrix (ECM) and cytoskeletal elements can contribute significantly as well [43,44]. In *Abcd1^−/y^* mice, activated SGC further alter neuronal membrane mechanical properties, thereby impacting MSC gating.

Limitations in our interpretation may arise from the role of other pathways identified by transcriptomic analysis. Many of the potassium channels upregulated in *Abcd1^−/y^* DRG, such as *Kcnj11* (protein Kir6.2) and *Kcnd2* (protein KV4.2), can be involved in peripheral pain–related pathways [26,45,46,47]. In addition to changes in ion transporters, various neuropeptides, such as galanin and neuropeptide Y, were downregulated in *Abcd1^−/y^* DRG. Galanin, a 29-aa peptide, is present in a small population of DRG neurons [48,49] and may influence nociception at different levels of the nervous system, including DRG, the spinal cord and brain [50,51].

## 5. Conclusions

We report that the pain syndrome seen in the myeloneuropathy of X-ALD arises from ABCD1 protein dysfunction in DRGs (Graphic abstract). Mice deficient in ABCD1 protein experience an early mechanical allodynia mediated by PIEZO2 dysfunction in DRG neurons. After blocking PIEZO2, a partial rescue of mechanical hypersensitivity occurs, but high levels of saturated VLCFA persist. These lipids accumulate specifically in SGCs, a metabolically active cell type surrounding DRG neurons. Our study thus points to dysregulated neuron–glia interactions in X-ALD and elucidates novel mechanisms involved in the genesis of mechanical allodynia and sensory pathology.

## Figures and Tables

**Table 1:** Enrichment analysis by process network of differential expressed genes between WT and *Abcd1^−/y^* DRG in RNA-seq using metacore bioinformatics tool.

#	Networks	*p*-Value	Network Objects
1	Muscle contraction	1.601 × 10^−4^	GALR2, K(+) channel, subfamily J, EDNRA, Galpha(i)-specific peptide GPCRs, MyHC, nAChR alpha, Galanin, Galpha(q)-specific peptide GPCRs
2	Inflammation_Interferon signaling	3.178 × 10^−3^	TIMP1, IRF7, IFI56, IL21R, IFI44
3	Signal transduction_Neuropeptide signaling pathways	1.326 × 10^−2^	NPY, Galpha(i)-specific peptide GPCRs, Secretogranin V, Galanin, Galpha(q)-specific peptide GPCRs
4	Signal transduction_Leptin signaling	1.619 × 10^−2^	NPY, Kir6.2, TIMP1, Galanin
5	Development_Blood vessel morphogenesis	1.695 × 10^−2^	Tissue kallikreins, EDNRA, Galpha(i)-specific peptide GPCRs, RBP-J kappa (CBF1), Galpha(q)-specific peptide GPCRs, EDNRB
6	Cardiac development_FGF_ErbB signaling	2.625 × 10^−2^	Kv4.2 channel, EDNRA, MyHC, BARX2
7	Transport_Potassium transport	3.153 × 10^−2^	K(+) channel, subfamily J, Kir6.2, Kv4.2 channel, SLC24A4, SERT
8	Apoptosis_Apoptotic mitochondria	3.513 × 10^−2^	Bcl-G, HSP70, HRK
9	Proliferation_Positive regulation cell proliferation	5.056 × 10^−2^	EDNRA, Galpha(i)-specific peptide GPCRs, TIMP1, ALK, CCKBR
10	Protein folding_ER and cytoplasm	6.076 × 10^−2^	HSC70, HSP70

**Table 2:** Enrichment analysis by process network of differentially expressed genes between WT and *Abcd1^−/y^* SGCs in RNA-seq using metacore bioinformatics tool.

#	Networks	*p*-Value	Network Objects
1	Cell adhesion_Amyloid proteins	1.453 × 10^−5^	Frizzled, NOTCH3 (3ICD), SFRP4, WNT11, NOTCH3, FZD4, SR-BI, Notch, Actin cytoskeletal, WNT, Caveolin-1, Actin
2	Development_Blood vessel morphogenesis	2.888 × 10^−4^	Galpha(q)-specific amine GPCRs, Galpha(i)-specific amine GPCRs, Galpha(i)-specific peptide GPCRs, FOXC1/2, PF4, Alpha-1B adrenergic receptor, ANGPTL4, Notch, Galpha(q)-specific peptide GPCRs, FOXC1, Transferrin
3	Development_Ossification and bone remodeling	1.463 × 10^−3^	Frizzled, Follistatin, SFRP4, FOXC1/2, FOXC1, IGF-1, IBP, WNT
4	Signal transduction_ Androgen receptor signaling cross-talk	3.145 × 10^−3^	Frizzled, IL-6, IGF-1, WNT, Caveolin-1
5	Cell adhesion_Cadherins	3.809 × 10^−3^	WISP1, Frizzled, WISP2, SFRP4, Actin cytoskeletal, WNT, F-Actin, Actin
6	Proliferation_Negative regulation of cell proliferation	3.938 × 10^−3^	IBP2, WISP2, Galpha(i)-specific peptide GPCRs, IBP5, IL-6, GPNMB (Osteoactivin), IGF-1, IBP
7	Cell adhesion_ Glycoconjugates	7.156 × 10^−3^	Fibulin-1, PF4, Elastin, HP, Actin cytoskeletal, Neurocan, Actin
8	Cell adhesion_Cell-matrix interactions	8.849 × 10^−3^	WISP1, MMP-12, Fibulin-1, Elastin, ITGA11, Mindin, LAMA1, Neurocan
9	Signal transduction_WNT signaling	1.137 × 10^−2^	WISP1, Frizzled, WISP2, SFRP4, WNT11, FOXC1, WNT
10	Development_Regulation of angiogenesis	1.152 × 10^−2^	Ephrin-A receptors, Galpha(i)-specific peptide GPCRs, Leptin receptor, AGTR2, PF4, IL-6, ANGPTL4, Galpha(q)-specific peptide GPCRs
11	Development_Skeletal muscle development	1.436 × 10^−2^	Actin muscle, Elastin, ITGA11, ACTG2, IGF-1, Actin
12	Development_Neurogenesis_Axonal guidance	1.439 × 10^−2^	Ephrin-A receptor 3, Ephrin-A receptors, NckAP1, PACAP receptor 1, Mindin, Actin cytoskeletal, DARPP-32, Actin
13	Signal transduction NOTCH signaling	1.620 × 10^−2^	Frizzled, NOTCH3 (3ICD), SFRP4, WNT11, NOTCH3, FZD4, WNT, PDGF-B
14	Development_Neurogenesis in general	1.761 × 10^−2^	Frizzled, Galpha(q)-specific amine GPCRs, Galpha(i)-specific amine GPCRs, NOTCH3, FZD4, Notch, WNT
15	Cell adhesion_Integrin-mediated cell-matrix adhesion	7.696 × 10^−2^	RHG7, Caveolin-2, ITGA11, Actin cytoskeletal, Caveolin-1, Actin

## Data Availability

Not applicable.

## Supplementary Materials

The following supporting information can be downloaded at: https://www.mdpi.com/article/10.3390/cells11111842/s1. Supplementary Table S1: Primer sequences used in qPCR analysis.; Supplementary Table S2: Enrichment analysis by toxicity network of differential expressed genes between WT and *Abcd1^−/y^* DRG in RNA-seq using metacore bioinformatics tool.; Supplementary Table S3: Enrichment analysis by GO processes of differential expressed genes between WT and *Abcd1^−/y^* DRG in RNA-seq using metacore bioinformatics tool.; Supplementary Table S4: Enrichment analysis by toxicity network of differential expressed genes between WT and *Abcd1^−/y^* SGCs in RNA-seq using metacore bioinformatics tool.; Supplementary Table S5: Enrichment analysis by GO processes of differential expressed genes between WT and *Abcd1^−/y^* SGCs in RNA-seq using metacore bioinformatics tool.; Supplementary Figure S1. Expression of ABCD family members (ABCD1, ABCD2, ABCD3) in DRG tissue compared to other nervous system at different ages. (A), Representative Western blot image showing ABCD family member expression in different nervous system tissues. Quantification of ABCD2 (B) and ABCD3 (C) protein levels in different nervous system tissues with GADPH as loading control. Results were expressed as means ± SEM. (*n* = 3 for each group).; Supplementary Figure S2. ABCD1 immunostaining in DRG from WT mouse at different ages including embryo 13.5(E13.5), postnatal day 8 (P8) and 2 months (2M) of age.; Supplementary Figure S3. Von Frey testing of *Abcd1^−/y^* mice by different technicians over the years show overall reproducibility of results across several generations of mice. (A) Mechanical sensitivity of *Abcd1^−/y^* mice measured by Von Frey testing in 2014 (*n* = 11 for WT and *n* = 14 for *Abcd1^−/y^).* (B) Mechanical sensitivity of *Abcd1^−/y^* mice measured by Von Frey testing in 2015 (*n* = 8 for WT and *n* = 8 for *Abcd1^−/y^).* Results were expressed as means ± SEM. * *p* < 0.05 and ** *p* < 0.01 as compared with WT control.; Supplementary Figure S4. Confocal image showing cleaved-caspase 3 staining (green) and NF (red) co-staining in DRG tissue from WT and *Abcd1^−/y^* mice at 15 months of age.; Supplementary Figure S5. Transcriptomic RNA-seq of *Abcd1^−/y^* mouse DRG at 13 months of age (*n* = 3 for both WT and *Abcd1^−/y^*). (A) Volcano plot showing the differentially expressed genes between WT and *Abcd1^−/y^* DRG. Heat map showing the hierarchical clustering of upregulated genes (B) and downregulated genes (C) in *Abcd1^−/y^* DRG at |log2 FC| > 1 threshold, indicating clean segregation between the WT and *Abcd1^−/y^* group.; Supplementary Figure S6. Bar graph showing the 80 upregulated genes (A) and 81 downregulated genes (B) in *Abcd1^−/y^* DRG at |log2 FC| > 1 threshold in the order from high to low. Genes selected for qPCR verification in Figure 5 were labeled with arrow.; Supplementary Figure S7. SGC/neuron ratio in *Abcd1^−/y^* DRG. (A) Representative images showing the SGC/neuron ratio in *Abcd1^−/y^* mouse DRG (Toluidine staining of 1 micron slices). *C* Quantification of SGC/neuron ratio from images (*n* = 4 for WT and *n* = 6 for *Abcd1^−/y^*). Results were expressed as means ± SEM.; Supplementary Figure S8. Transcriptomic RNA-seq analysis of primary cultured SGCs of *Abcd1^−/y^* mice at 1 month of age (*n* = 3 for WT and *n* = 2 for *Abcd1^−/y^*). (A) Volcano plot showing the differentially expressed genes between WT and *Abcd1^−/y^* SGCs. Bar graph showing the 85 upregulated genes (B) and 111 downregulated genes (C) in *Abcd1^−/y^* SGCs at |log2 FC| > 1 threshold in the order from high to low.
