# Supplementary material for: Peroxisome Metabolism Contributes to PIEZO2-Mediated Mechanical Allodynia
Source: Cells. 2022 Jun 4;11(11):1842. doi: 10.3390/cells11111842 (PMC9180358; doi:10.3390/cells11111842)
Supplement: Supplementary file 1 [file cells-11-01842-s001.zip › cells-1708087-supplementary/Table S1.pdf]

Table S1 qPCR Primers

Gene	Forward primer	Reverse primer	Amplicon
Kcnj11	aagggcattatccctgaggaa	ttgcctttcttggacacgaag	110bp
Slc24a4	ggaccggaaactgggcatc	tgggcaagttgacgaaggtaa	101bp
Kcnd2	gggtggatgcctgttgctt	gtcttgccatgtctggaaacg	120bp
Galanin	ggcagcgttatcctgctagg	ctgttcagggtccaacctct	104bp
Galr2	gcggtcctcgtacccctatt	cacaggatgaaacacaggtcg	158bp
Gpr179	cactaccgtcccaagccaag	tgacagccgttgaacatctcc	107bp
Npy	atgctaggtaacaagcgaatgg	tgtcgcagagcggagtagtat	161bp
Gfap	cggagacgcatcacctctg	agggagtggaggagtcattcg	126bp
mme	ctctctgtgcttgtcttgctc	gacgttgcgtttcaaccagc	198bp
Cav-1	atgtctgggggcaaatacgtg	cgcgtcatacacttgcttct	132bp
Cav-2	tcaccagctcaactctcatct	gccagaaatacggtcaggaact	153bp
Lama1	cagcgccaatgctacctgt	ggattcgtactgttaccgtcaca	123bp
Eln	ttgctgatcctcttgctcaac	gcccctggataatagactccac	113bp
Ncan	tgcaaccacggctaagctc	ggggataagcaggcaatgac	165bp
Piezo1	ccctcaagacacagcatacca	tgcccaaaggttacagttttgtcc	159bp
Piezo2	cagtaggacacacagggcg	gttgagcagttgtaagcggg	139bp
b-Actin	acaaccttcttgcagctcctc	ctgacccatacccaccatcac	200bp
