# Supplementary material for: Peroxisome Metabolism Contributes to PIEZO2-Mediated Mechanical Allodynia
Source: Cells. 2022 Jun 4;11(11):1842. doi: 10.3390/cells11111842 (PMC9180358; doi:10.3390/cells11111842)
Supplement: Supplementary file 1 [file cells-11-01842-s001.zip › cells-1708087-supplementary/Table S2.pdf]

Table S2 Enrichment by Toxicity Network

#	Networks	p-value	Network Objects
1	Signal transduction_Signal transducer and activator of transcription 1, 91kDa	9.973E-04	HLA-DRA1, TIMP1, IRF7, PDGF-B
2	Signal transduction_Neuropeptide signaling pathway	3.851E-03	NPY, Galpha(i)-specific peptide GPCRs, Galanin
3	Protein folding_p53 regulation	1.253E-02	HSC70, HSP70
4	Transmission of nerve impulse_Opioid, galanin and neurotensin receptors	1.253E-02	GALR2, Galpha(i)-specific peptide GPCRs
5	Protein folding_ATFs regulation	1.492E-02	V1a receptor, Galpha(i)-specific peptide GPCRs, Galpha(q)-specific peptide GPCRs
6	Protein folding_HSP70 regulation by HSFs	2.086E-02	HSC70, HSP70
7	Inflammation_Interferon signaling	3.907E-02	TIMP1, IRF7
8	Immune response_CCL20	4.611E-02	IRAK4, TLR6
9	Development_Cytoskeleton biogenesis	4.979E-02	VDB, MyHC
