# Supplementary material for: Peroxisome Metabolism Contributes to PIEZO2-Mediated Mechanical Allodynia
Source: Cells. 2022 Jun 4;11(11):1842. doi: 10.3390/cells11111842 (PMC9180358; doi:10.3390/cells11111842)
Supplement: Supplementary file 1 [file cells-11-01842-s001.zip › cells-1708087-supplementary/Table S3.pdf]

Table S3 Enrichment by Go Process

#	Processes	p-value
1	response to interferon-beta	7.276E-16
2	cellular response to interferon-beta	2.699E-15
3	positive regulation of response to external stimulus	8.819E-11
4	positive regulation of defense response	1.698E-10
5	regulation of defense response	3.592E-10
6	response to external stimulus	5.960E-10
7	regulation of multi-organism process	7.743E-10
8	defense response to virus	1.163E-09
9	regulation of response to external stimulus	2.280E-09
10	cellular response to cytokine stimulus	3.222E-09
11	regulation of response to biotic stimulus	4.836E-09
12	positive regulation of multi-organism process	4.836E-09
13	positive regulation of response to biotic stimulus	5.665E-09
14	response to virus	6.766E-09
15	response to biotic stimulus	7.654E-09
16	response to other organism	1.335E-08
17	response to external biotic stimulus	1.402E-08
18	immune system process	1.516E-08
19	response to cytokine	1.696E-08
20	regulation of immune system process	2.034E-08
21	regulation of large conductance calcium-activated potassium channel activity	3.018E-08
22	positive regulation of large conductance calcium-activated potassium channel activity	3.018E-08
23	regulation of response to stress	3.341E-08
24	defense response	4.582E-08
25	regulation of immune response	4.895E-08
26	positive regulation of interleukin-1 beta production	6.504E-08
27	positive regulation of somatostatin secretion	7.006E-08
28	positive regulation of innate immune response	7.809E-08
29	defense response to other organism	1.352E-07
30	regulation of viral genome replication	1.535E-07
31	positive regulation of immune response	1.901E-07
32	regulation of interleukin-1 beta production	2.477E-07
33	cellular response to organic substance	2.827E-07
34	positive regulation of immune system process	2.857E-07
35	multi-organism process	3.524E-07
36	response to organic substance	4.513E-07
37	positive regulation of interleukin-1 production	4.792E-07
38	regulation of somatostatin secretion	6.467E-07
39	regulation of innate immune response	8.970E-07
40	negative regulation of viral genome replication	9.328E-07
41	copulation	1.165E-06
42	endothelin receptor signaling pathway	1.216E-06
43	galanin-activated signaling pathway	1.216E-06
44	cholecystokinin signaling pathway	1.216E-06
45	extracellular matrix disassembly	1.433E-06
46	positive regulation of response to stimulus	1.633E-06
47	positive regulation of ion transport	1.671E-06
48	activation of immune response	1.772E-06
49	response to stress	1.800E-06

50	regulation of interleukin-1 production	1.869E-06
