# Supplementary material for: Peroxisome Metabolism Contributes to PIEZO2-Mediated Mechanical Allodynia
Source: Cells. 2022 Jun 4;11(11):1842. doi: 10.3390/cells11111842 (PMC9180358; doi:10.3390/cells11111842)
Supplement: Supplementary file 1 [file cells-11-01842-s001.zip › cells-1708087-supplementary/Table S4.pdf]

Table S4 Enrichment by Go Process

#	Processes	p-value
1	response to organic cyclic compound	1.130E-23
2	response to oxygen-containing compound	1.019E-22
3	circulatory system development	6.559E-22
4	response to organic substance	2.703E-20
5	response to hormone	4.769E-20
6	tube development	1.400E-19
7	tissue development	1.830E-19
8	response to lipid	1.912E-19
9	cardiovascular system development	9.926E-19
10	vasculature development	2.686E-18
11	positive regulation of response to stimulus	9.099E-18
12	positive regulation of multicellular organismal process	1.014E-17
13	regulation of multicellular organismal process	1.697E-17
14	blood vessel development	1.789E-17
15	tube morphogenesis	2.415E-17
16	tissue remodeling	6.181E-17
17	positive regulation of developmental process	1.055E-16
18	response to endogenous stimulus	1.071E-16
19	regulation of cell population proliferation	1.200E-16
20	animal organ development	1.517E-16
21	response to external stimulus	1.652E-16
22	response to drug	1.882E-16
23	regulation of response to stimulus	4.865E-16
24	negative regulation of multicellular organismal process	6.675E-16
25	regulation of developmental process	9.371E-16
26	anatomical structure morphogenesis	9.384E-16
27	response to chemical	9.497E-16
28	blood vessel morphogenesis	1.101E-15
29	system development	1.151E-15
30	response to other organism	1.380E-15
31	response to external biotic stimulus	1.497E-15
32	negative regulation of cell population proliferation	1.942E-15
33	multicellular organism development	2.435E-15
34	mesenchyme morphogenesis	2.852E-15
35	regulation of molecular function	3.163E-15
36	response to biotic stimulus	3.755E-15
37	regulation of locomotion	4.700E-15
38	tissue morphogenesis	7.252E-15
39	muscle structure development	7.488E-15
40	regulation of response to stress	8.091E-15
41	heart development	8.602E-15
42	positive regulation of locomotion	1.104E-14
43	anatomical structure formation involved in morphogenesis	1.486E-14
44	regulation of MAPK cascade	1.694E-14
45	regulation of multicellular organismal development	2.047E-14
46	regulation of cell motility	2.395E-14
47	response to nitrogen compound	2.845E-14
48	positive regulation of cell differentiation	3.043E-14
49	response to organonitrogen compound	3.335E-14

50	animal organ morphogenesis	4.448E-14
