# Supplementary material for: Peroxisome Metabolism Contributes to PIEZO2-Mediated Mechanical Allodynia
Source: Cells. 2022 Jun 4;11(11):1842. doi: 10.3390/cells11111842 (PMC9180358; doi:10.3390/cells11111842)
Supplement: Supplementary file 1 [file cells-11-01842-s001.zip › cells-1708087-supplementary/Table S5.pdf]

Table S5 Enrichment by Toxicity Network

#	Networks	p-value	Network Objects
1	Transport_Lipid transport	2.144E-04	CD36, HDL proteins, SR-BI, Caveolin-1
2	Transport_Vesicle-mediated transport	3.372E-03	VDR, SR-BI, CD14
3	Cell adhesion_Cytoskeleton regulation through CDC42	5.264E-03	RHG7, Actin cytoskeletal, Actin
4	Cell adhesion_Cytoskeleton regulation through RhoA	5.818E-03	RHG7, Actin cytoskeletal, Actin
5	Cell adhesion_Integrins signaling to Beta-catenin	7.003E-03	Frizzled, Actin cytoskeletal, WNT, Actin
6	Development_Skeletal development-Osteopontin signaling	7.030E-03	VDR, Actin cytoskeletal, Actin
7	Blood coagulation_Fibrinogen signaling	7.030E-03	IL-6, Actin cytoskeletal, Actin
8	Cell adhesion_PTP-1B	8.322E-03	Actin cytoskeletal, Actin
9	Protein folding_ATFs regulation	1.199E-02	CCX CKR, Galpha(i)-specific peptide GPCRs, AGTR2, Galpha(q)-specific peptide GPCRs
10	Cell adhesion_p120-catenin	1.343E-02	Actin cytoskeletal, Actin
11	Cell adhesion_Collagen III_Actin	1.343E-02	Actin cytoskeletal, Actin
12	Transmission of nerve impulse_Ephrin receptors	1.524E-02	Ephrin-A receptor 3, Ephrin-A receptors, Actin
13	Signal transduction_Wnt receptor signaling pathway	1.538E-02	Frizzled, WNT
14	Cell adhesion_E-cadherin	1.743E-02	Actin cytoskeletal, Actin
15	Metabolism_Histamine_ABP1	1.959E-02	AL1A1, AOC3
16	Signal transduction_IL-6R signaling_haptoglobin	2.423E-02	IL-6, HP
17	Cell adhesion_Actin cytoskeleton	2.451E-02	GPX3, Actin cytoskeletal, Actin
18	Inflammation_IL-6 signaling through gp130	3.008E-02	IL-6, HP, Transferrin
19	Inflammation_GRO-gamma signaling	3.156E-02	IL-6, Actin cytoskeletal, Actin
20	Inflammation_Annexin A1 signaling	3.156E-02	IL-6, Actin cytoskeletal, Actin
21	Metabolism_Lipid metabolism	3.309E-02	APOC1, LCAT, HDL proteins
22	Cell adhesion_PAI1 signaling, PPAR-beta (delta), RXR-alpha regulation	3.465E-02	Frizzled, WNT, Caveolin-1
23	Cell adhesion_Cadherins	3.755E-02	Actin cytoskeletal, Actin
24	Cell adhesion_Chemokine receptor signaling_integrin alpha-L	3.957E-02	Actin cytoskeletal, CD80, Actin
25	Inflammation_Signaling to STAT3	4.128E-02	Leptin receptor, IL-6, PDGF-B
26	Chemotaxis_Fibronectin, VEGF-A, Thrombopoietin, ENA-78 signaling	4.128E-02	IL-6, Actin cytoskeletal, Actin
27	Cell adhesion_Collagen I signaling	4.128E-02	VDR, Actin cytoskeletal, Actin
28	Inflammation_CXCR4 signaling	4.128E-02	Galpha(i)-specific peptide GPCRs, Actin cytoskeletal, Actin
29	Cell adhesion_G protein alpha 12 signaling	4.303E-02	IL-6, Actin cytoskeletal, Actin
30	Inflammation_IL-8 signaling	4.303E-02	Galpha(i)-specific peptide GPCRs, IL-6, Actin
31	Inflammation_C-reactive protein	4.351E-02	Leptin receptor, IL-6
32	Metabolism_Aldehyde metabolism	4.351E-02	AL1A1, AOC3

33	Metabolism_Dopamine_ABP-1	4.351E-02	AL1A1, AOC3
34	Development_Osteoblast differentiation	4.482E-02	VDR, Actin cytoskeletal, Actin
35	Signal transduction_STAT6	4.661E-02	Leptin receptor, PDGF-B
