# Supplementary figures and images for: Peroxisome Metabolism Contributes to PIEZO2-Mediated Mechanical Allodynia
Source: Cells. 2022 Jun 4;11(11):1842. doi: 10.3390/cells11111842 (PMC9180358; doi:10.3390/cells11111842)

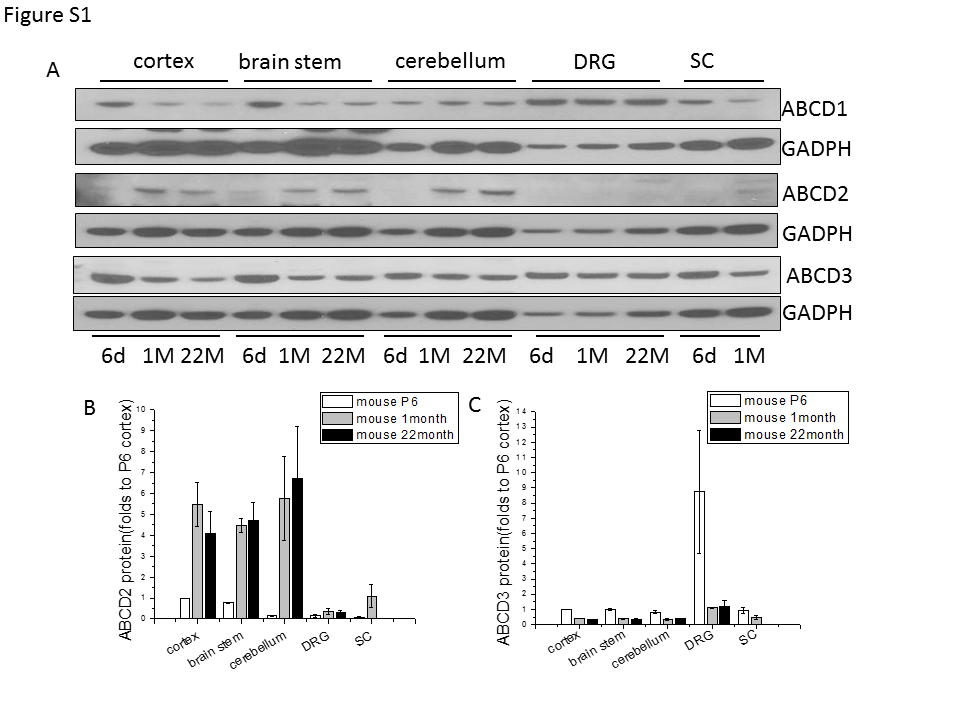

Supplement: Supplementary file 1 [file cells-11-01842-s001.zip › cells-1708087-supplementary/FigureS1.TIF]

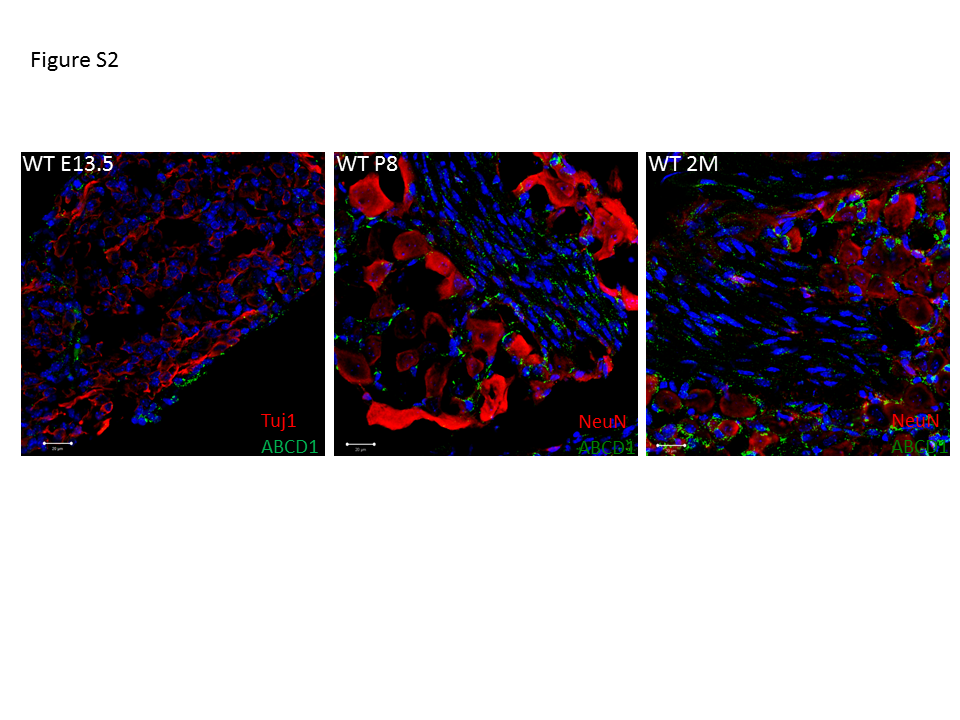

Supplement: Supplementary file 1 [file cells-11-01842-s001.zip › cells-1708087-supplementary/FigureS2.TIF]

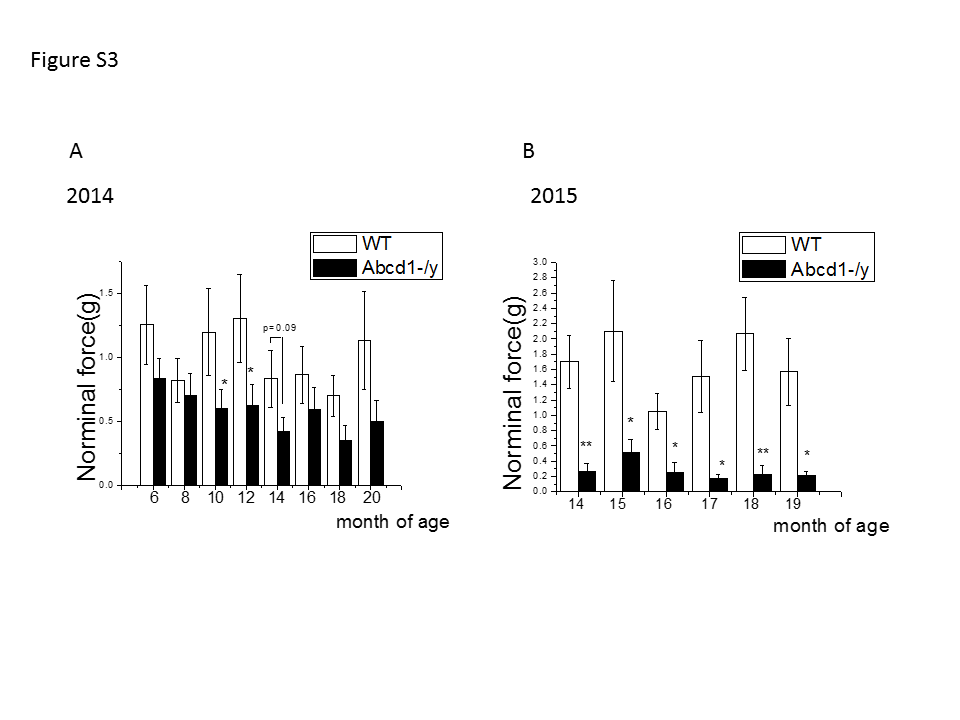

Supplement: Supplementary file 1 [file cells-11-01842-s001.zip › cells-1708087-supplementary/FigureS3.TIF]

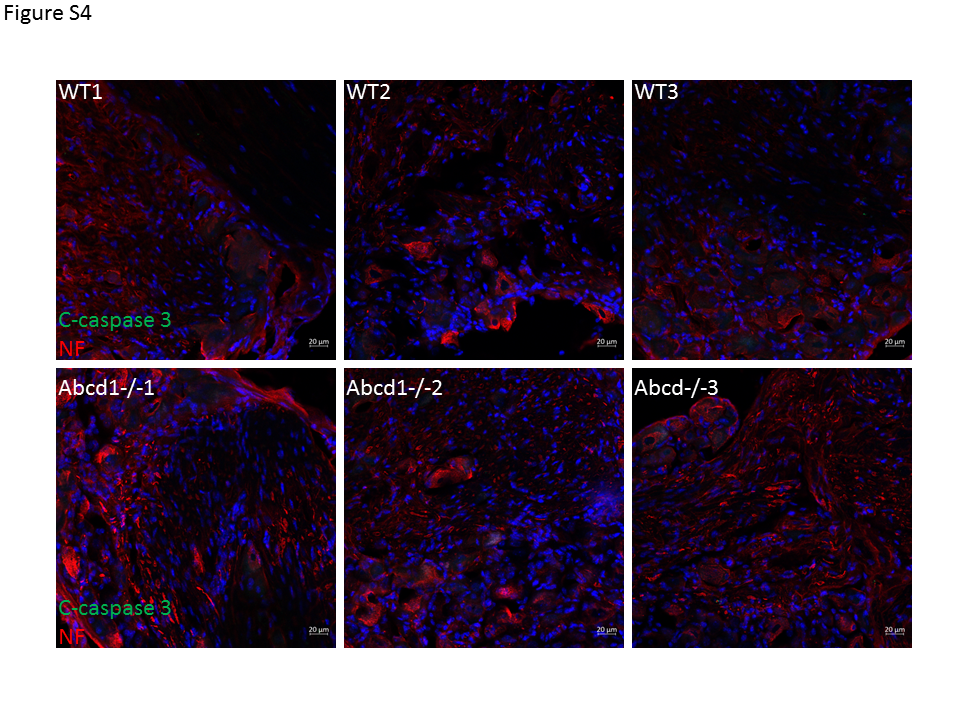

Supplement: Supplementary file 1 [file cells-11-01842-s001.zip › cells-1708087-supplementary/FigureS4.TIF]

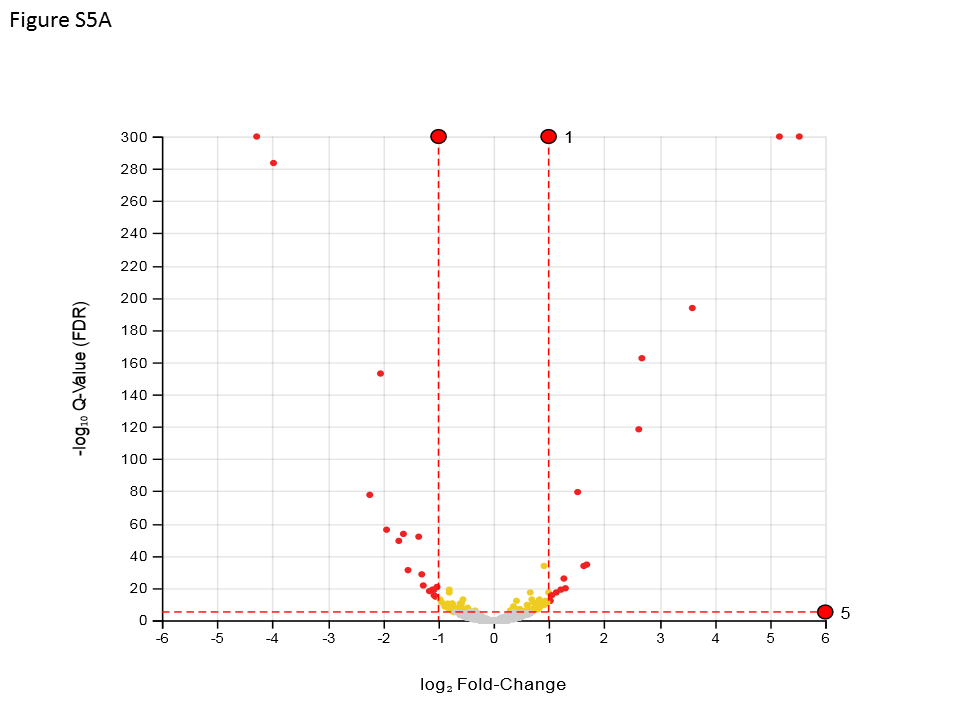

Supplement: Supplementary file 1 [file cells-11-01842-s001.zip › cells-1708087-supplementary/FigureS5A.TIF]

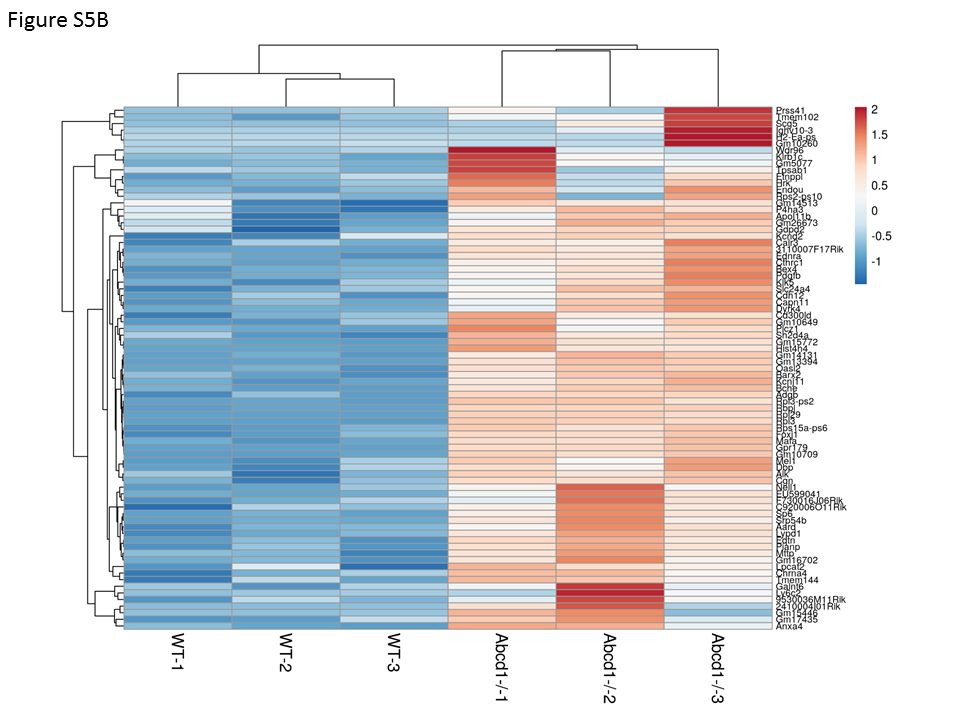

Supplement: Supplementary file 1 [file cells-11-01842-s001.zip › cells-1708087-supplementary/FigureS5B.TIF]

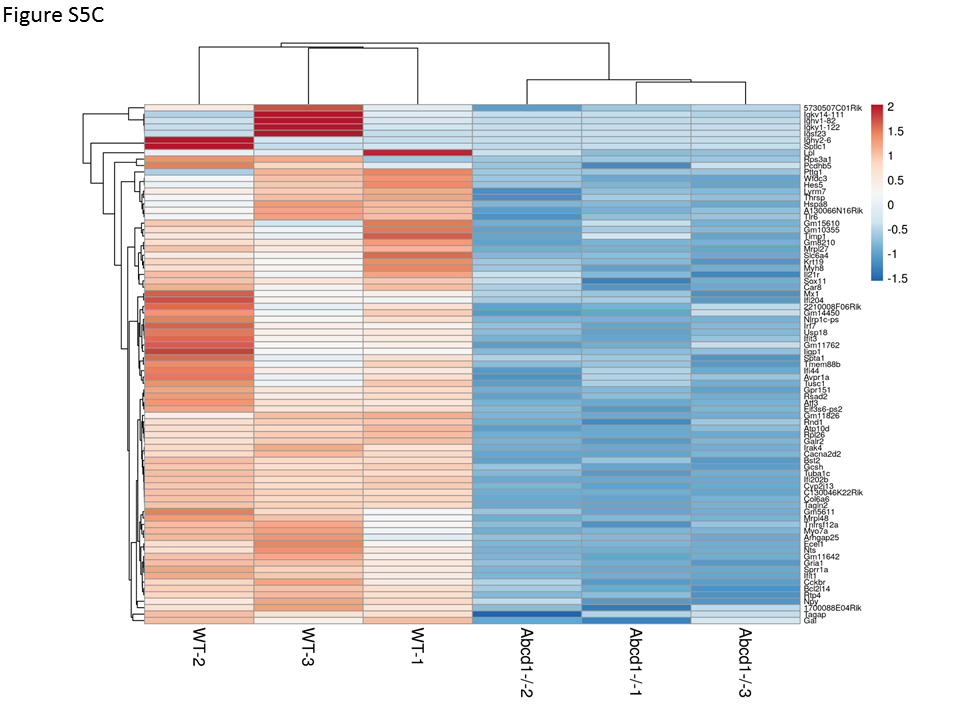

Supplement: Supplementary file 1 [file cells-11-01842-s001.zip › cells-1708087-supplementary/FigureS5C.TIF]

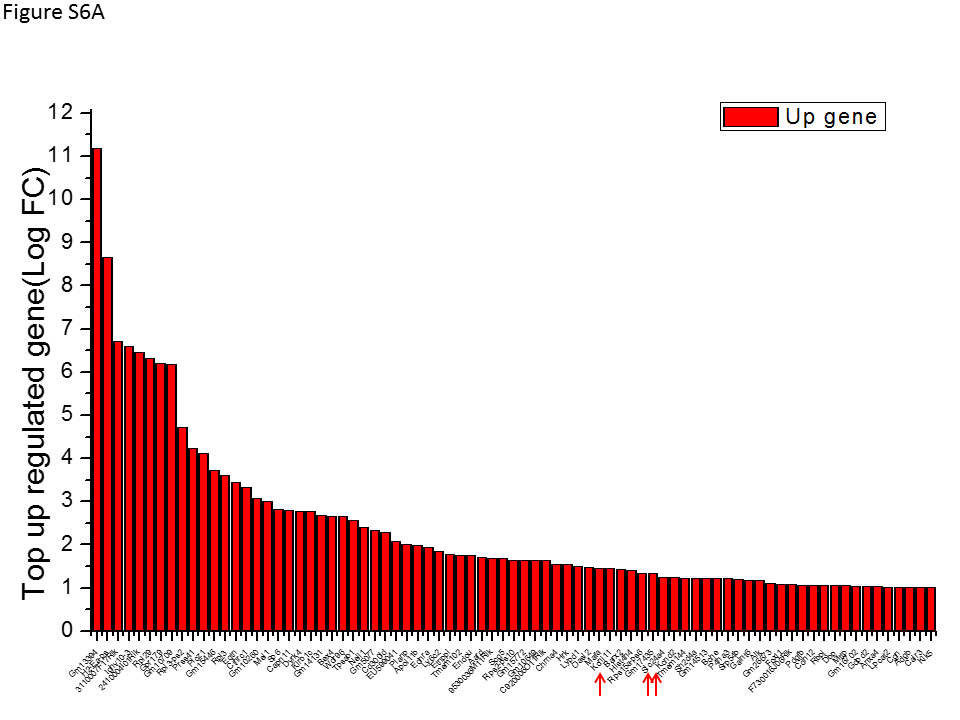

Supplement: Supplementary file 1 [file cells-11-01842-s001.zip › cells-1708087-supplementary/FigureS6A.TIF]

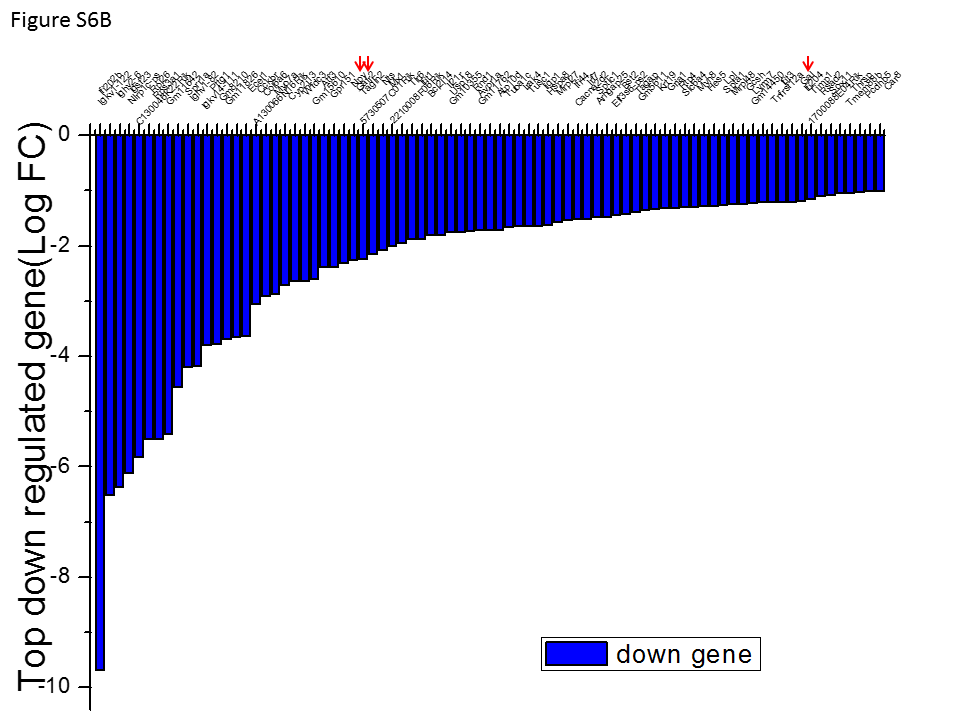

Supplement: Supplementary file 1 [file cells-11-01842-s001.zip › cells-1708087-supplementary/FigureS6B.TIF]

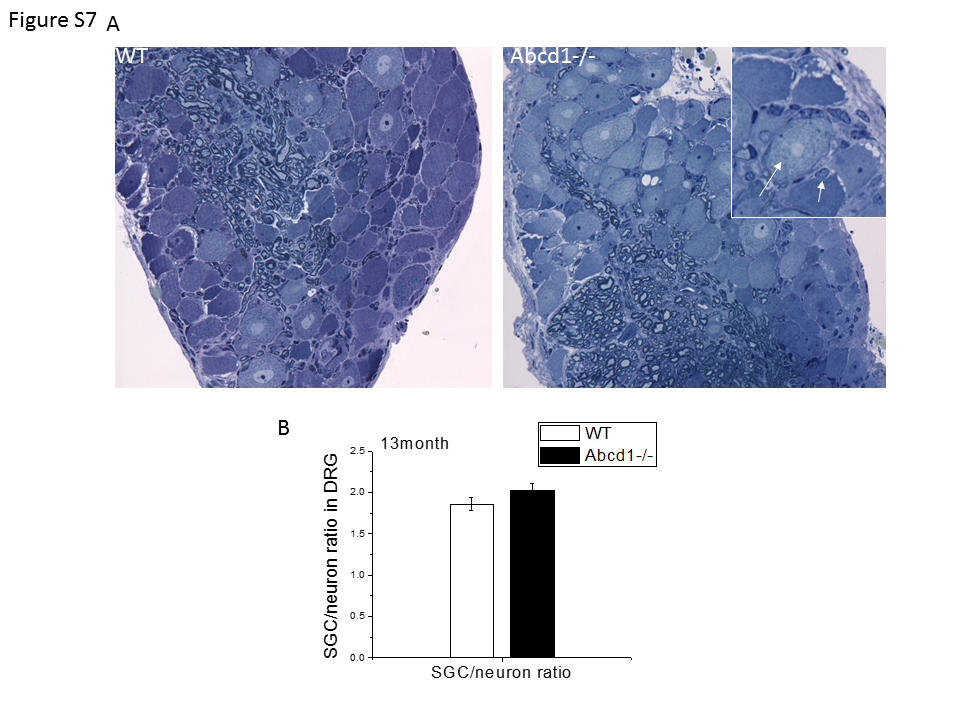

Supplement: Supplementary file 1 [file cells-11-01842-s001.zip › cells-1708087-supplementary/FigureS7.TIF]

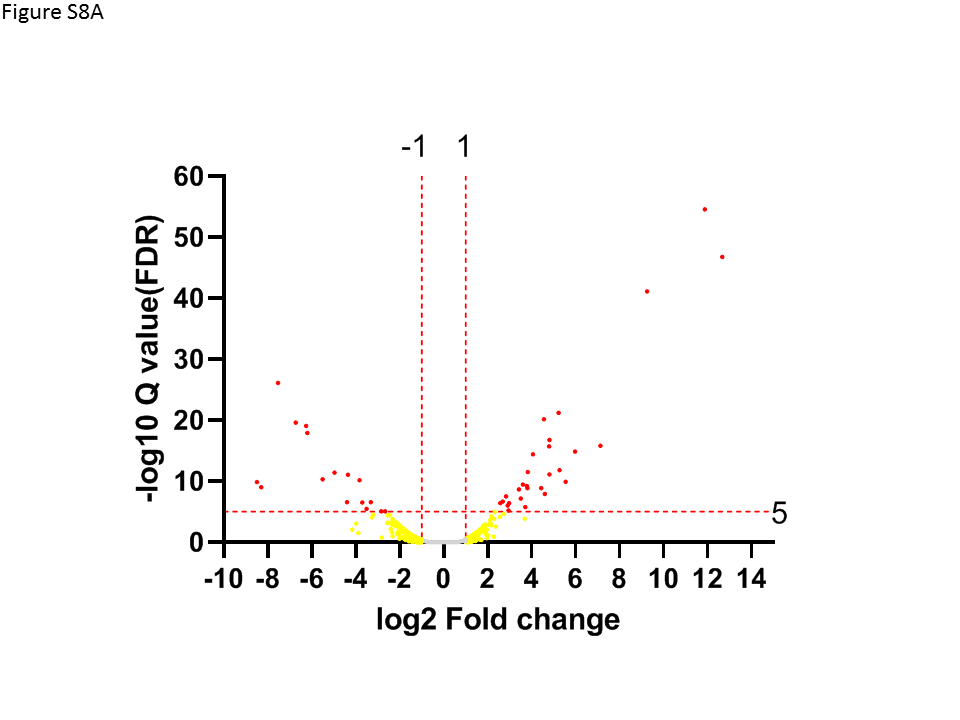

Supplement: Supplementary file 1 [file cells-11-01842-s001.zip › cells-1708087-supplementary/FigureS8A.TIF]

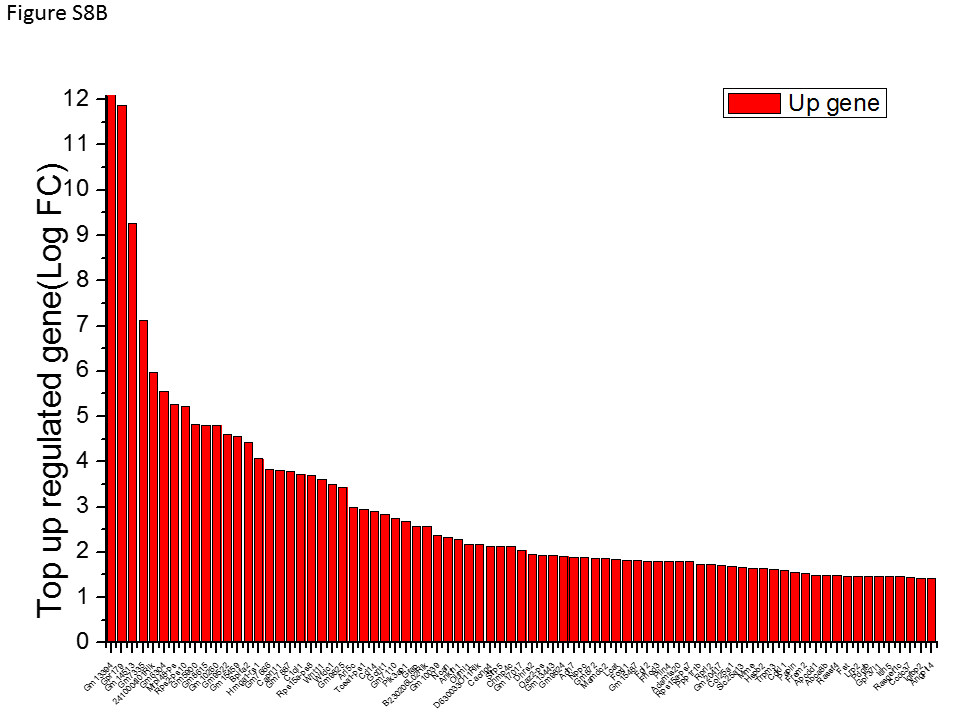

Supplement: Supplementary file 1 [file cells-11-01842-s001.zip › cells-1708087-supplementary/FigureS8B.TIF]

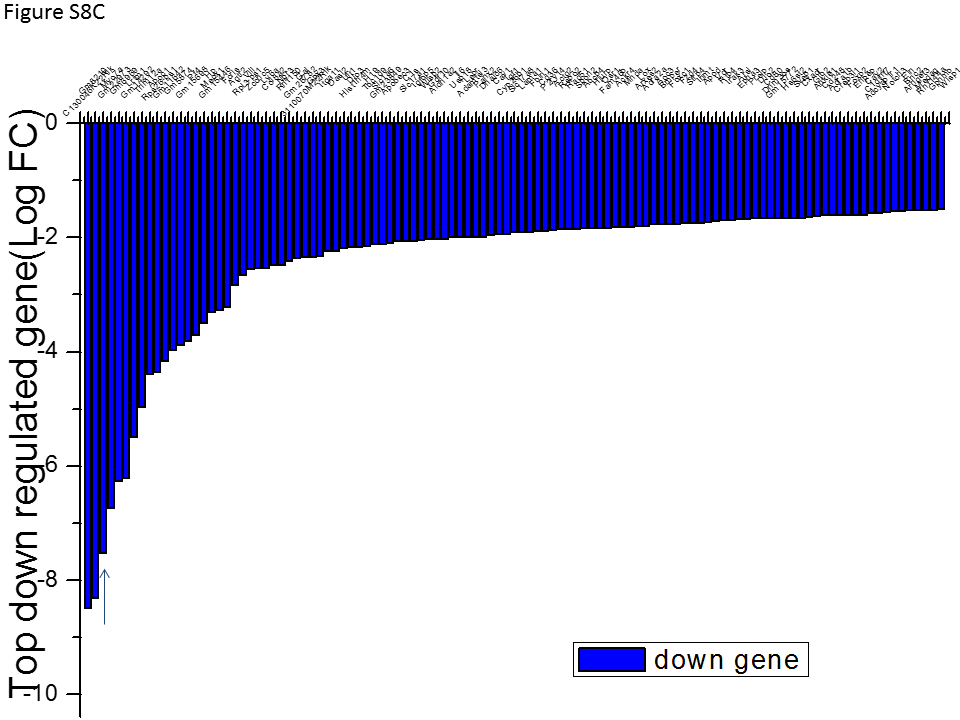

Supplement: Supplementary file 1 [file cells-11-01842-s001.zip › cells-1708087-supplementary/FigureS8C.TIF]
